# Highly Stable Supramolecular Donor–Acceptor Complexes Involving (*Z*)-, (*E*)-di(3-pyridyl)ethylene Derivatives as Weak Acceptors: Structure—Property Relationships

**DOI:** 10.3390/molecules30193920

**Published:** 2025-09-29

**Authors:** Artem I. Vedernikov, Valeriy V. Volchkov, Mikhail N. Khimich, Mikhail Y. Mel’nikov, Fedor E. Gostev, Ivan V. Shelaev, Victor A. Nadtochenko, Lyudmila G. Kuz’mina, Judith A. K. Howard, Asya A. Efremova, Mikhail V. Rusalov, Sergey P. Gromov

**Affiliations:** 1 NRC “Kurchatov Institute”, Kurchatov Complex of Crystallography and Photonics, Photochemistry Center, 119421 Moscow, Russia; 2 Department of Chemistry, M. V. Lomonosov Moscow State University, 119991 Moscow, Russia; 3 N. N. Semenov Federal Research Center for Chemical Physics, Russian Academy of Sciences, 119991 Moscow, Russia; 4 N. S. Kurnakov Institute of General and Inorganic Chemistry, Russian Academy of Sciences, 119991 Moscow, Russia; 5 Department of Chemistry, Durham University, Durham DH1 3LE, UK

**Keywords:** dipyridylethylenes, bis(crown)stilbene, supramolecular complexes, intrasupramolecular photo-driven electron transfer, X-ray diffraction, NMR spectroscopy, time-resolved absorption spectroscopy, TD-DFT calculations

## Abstract

The *Z*-isomer of *N*,*N*’-diammoniopropyl derivative of di(3-pyridyl)ethylene was synthesized. The structure and stability of complexes between this non-planar weak acceptor (A, (*Z*)-**2**) and a planar strong donor, the *E*-isomer of bis(18-crown-6)stilbene (D, (*E*)-**1**), were studied using X-ray diffraction, ^1^H NMR spectroscopy, and optical spectroscopy, including ^1^H NMR and spectrofluorimetric titrations. In MeCN, the components form a very stable pseudocyclic bimolecular complex (log*K*_D·A_ = 8.48) due to homoditopic coordination of the ammonium groups of the acceptor to the crown moieties of the donor through numerous hydrogen bonds. Intrasupramolecular photo-driven electron transfer (ET) in the isomeric complexes of (*E*)-**1** with (*E*)- and (*Z*)-**2** was studied using steady-state absorption and fluorescence spectroscopy with time-resolved pulse absorption spectroscopy. It was found that back ET is approximately two times faster in complex (*E*)-**1·**(*Z*)-**2** than in closely related (*E*)-**1·**(*E*)-**2**. Meanwhile, it is ~67 times slower in complex (*E*)-**1·**(*E*)-**2** than in the isomeric complex based on *N*,*N*’-diammoniopropyl derivative of (*E*)-di(4-pyridyl)ethylene. Quantum chemical (DFT, TD-DFT) calculations suggest the actual photorelaxation pathway for the complexes under study.

## 1. Introduction

**Scheme 1:**
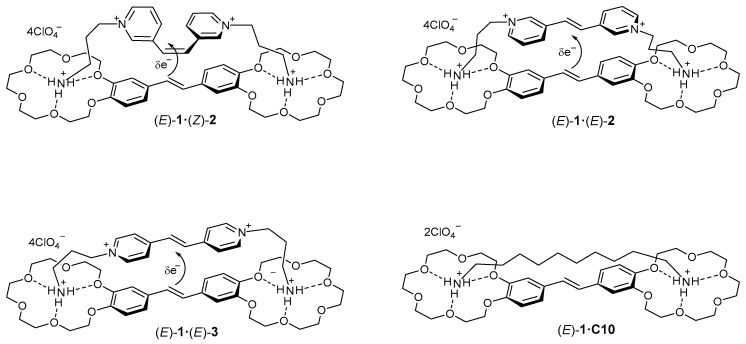
Structures of pseudocyclic 1:1 D–A complexes.

Organic charge transfer (CT) complexes has attracted the attention of researchers owing to their wide use in various fields of materials science and chemistry such as development of organic solar cells [1,2,3], electromagnetic materials [4], organic semiconductors [5], photocatalysts [6], and fluorescent sensors [7,8,9], design of artificial light-harvesting antennas [10,11], and molecular electronics [12]. The organic intra- and intermolecular charge transfer crown-containing complexes continue to attract the attention of researchers owing to diverse prospects for their practical use. Previously, we demonstrated the applicability of crown-containing unsaturated dyes and their complexes as selective chromo- and fluoroionophores, for the design of photochromic and fluorescent materials, as photochromic ionophores in the photocontrolled ion transport across membranes, in polymeric and photoswitchable Langmuir–Blodgett films, etc. [13,14]. For the present study, it is important that highly stable complexes based on bis-crown-containing unsaturated compounds bound by non-covalent interactions serve as models to study the dynamics of intrasupramolecular electron transfer (ET) [14,15]. Indeed, ultrafast photodynamics was found for bi- and termolecular stilbene–viologen complexes [16]. It was shown that the electron transfer efficiency can be controlled and the photoreaction pathway can be changed by varying the structures of the donor and acceptor components of the complex and excitation conditions [16,17]. A structural factor affecting the distance between D and A and, hence, the probability of the intrasupramolecular electron transfer is the number of methylene units in the *N*-ammonioalkyl substituents of the acceptor [18,19,20,21]. One more effective factor is the non-parallel arrangement of generally planar conjugated moieties of the donor and the acceptor attained as a result of structural asymmetry of the acceptor [22]. A shortened photoreaction pathway was implemented upon selective excitation of the D-A complex at the charge transfer band [16,17]. In this case, the CT* state of the complex is formed directly, while the intermediate states that are populated upon excitation of the major π–π* band of either donor or acceptor are bypassed. In all cases, the resulting CT* state undergoes back electron transfer during deactivation. Currently, no studies of the photo-driven electron transfer in any isomeric complexes with bis-crown derivatives are available from the literature. Therefore, it is of interest to elucidate the differences between the types of photo-driven electron transfer in D-A complexes between (*E*)-bis(18-crown-6)stilbene ((*E*)-**1**, strong donor) and *N*,*N*’-diammoniopropyl derivatives of *Z*- and *E*-isomers of viologen analog, di(3-pyridyl)ethylene ((*Z*)-**2** and (*E*)-**2**, weak acceptors), or isomeric viologen analog, di(4-pyridyl)ethylene (*E*)-**3** (strong acceptor, Scheme 1). The complexes are highly stable owing to hydrogen bonding. This masks the effect of intermolecular distance in D-A complexes. This paper describes the synthesis of compound (*Z*)-**2** and the study of the structure and stability of its supramolecular complexes with (*E*)-**1** by X-ray diffraction, ^1^H NMR spectroscopy, and electronic spectroscopy, including ^1^H NMR and competitive fluorimetric titration procedures.

The implemented approach to the study of intrasupramolecular photo-driven electron transfer in isomeric complexes (*E*)-**1·**(*Z*)-**2** and (*E*)-**1·**(*E*)-**2** includes the steady-state absorption and fluorescent spectroscopy and time-resolved absorption spectroscopy with femtosecond excitation in combination with quantum chemical data.

## 2. Results and Discussion

### 2.1. Synthesis

**Scheme 2:**
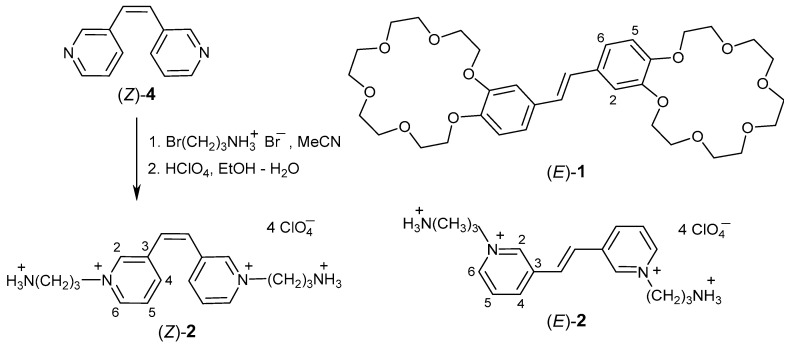
Synthesis of (*Z*)-**2**; structures of (*E*)-**1** and (*E*)-**2**.

*N*,*N*’-Di(ammoniopropyl) derivative (*Z*)-**2** was prepared by quaternization of (*Z*)-di(3-pyridyl)ethylene ((*Z*)-**4**) with excess 3-bromopropylammoium bromide in refluxing MeCN, followed by treatment with concentrated HClO_4_, which gave the tetraperchlorate in an overall yield of 74% (Scheme 2). Compound (*Z*)-**2** proved to be stable and did not transform into the *E*-isomer during quaternization and on long-term storage in the dark. The ^1^H and ^13^C NMR spectra in DMSO-*d*_6_ show the hydrogen and carbon signals of the central HC=CH group of compound (*Z*)-**2** at *δ* 7.13 and 128.71 ppm, respectively, while in the spectrum of (*E*)-**2**, analogous signals in the same solvent are located at *δ* 7.72 and 128.23 ppm (see Figures S1, S2, S4 and S5 in the Supplementary Materials to this article). Also, the signals of aromatic 2-H, 4-H, and 5-H atoms of (*Z*)-**2** are shifted upfield by 0.18–0.43 ppm compared to similar signals of (*E*)-**2**. This reflects pronounced twisting of the conjugated moiety in (*Z*)-**2** compared to the planar moiety in (*E*)-**2**. Consequently, the indicated hydrogen atoms of the *Z*-isomer go out of the deshielding regions of the neighboring benzene ring and ethylene bond.

The 1:1 supramolecular complex was obtained in the solid state by precipitation from a MeCN solution containing equimolar amounts of bis(18-crown-6)stilbene (*E*)-**1** and diammonium compound (*Z*)-**2** by slow saturation with benzene vapor. The formal stoichiometry of this complex was confirmed by ^1^H NMR spectroscopy (Figure S3 in Supplementary Materials), elemental analysis, and X-ray crystallography data. Since (*Z*)-**2** is a non-planar molecule and a weak acceptor, its complex with stilbene (*E*)-**1** is slightly yellowish, indicating very weak donor–acceptor interactions between the components. The related complex (*E*)-**1·**(*E*)-**2** is bright yellow in the solid state [18], which is indicative of more intense interactions between the planar conjugated moieties of the donor and acceptor components in the latter case.

### 2.2. Absorption and NMR Spectroscopy

**Scheme 3:**
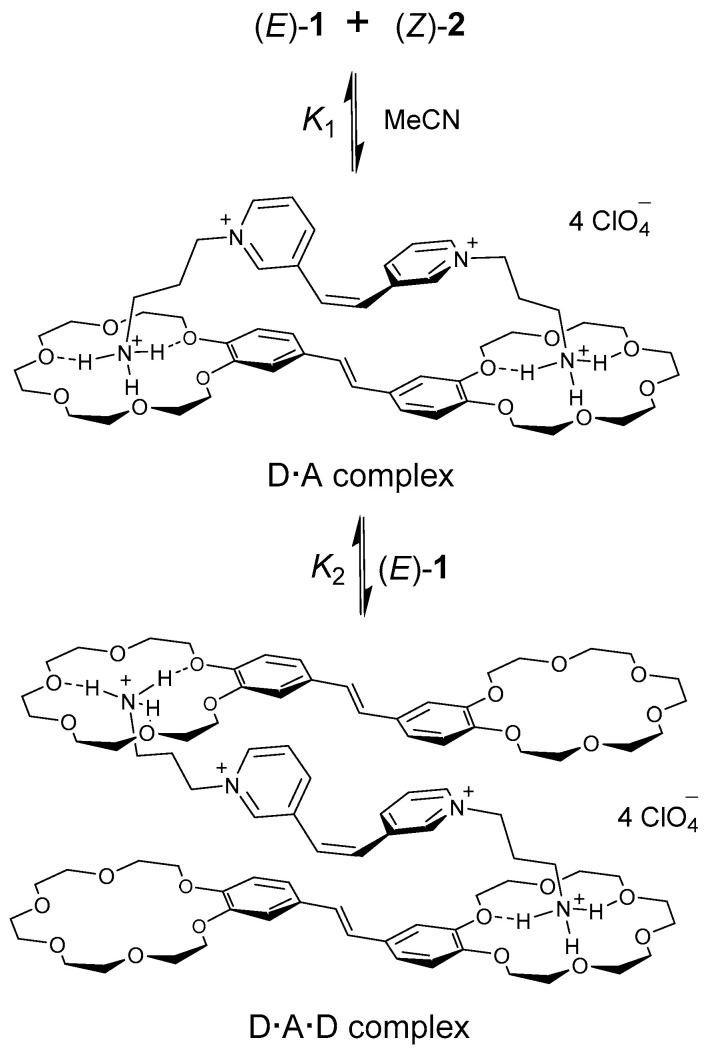
Formation of D·A and D·A·D complexes.

The complexes of weak acceptors (*Z*)-**2** and (*E*)-**2** with stilbene (*E*)-**1** are characterized by very high Δ*E*_CT_; therefore, the CT band is either manifested as a long-wavelength shoulder of the intense absorption band caused by local S_0_–S_1_ electron transitions (in the case of (*E*)-**2** [18]) or is fully covered by local transfer bands (in the case of (*Z*)-**2**). The weak electron-acceptor ability of compounds (*Z*)-**2** and (*E*)-**2** is a consequence of the non-planar chromophore structure in (*Z*)-**2** and, additionally, the relatively weak π-conjugation of the pyridine rings (linked by an ethylene bridge via the β-positions) in both cases. In our previous study considering systems based on (*E*)-**1** and diammonium-containing viologen analogs as acceptors (including (*E*)-**2** and (*E*)-**3**), we found [18,23,24] that MeCN solutions containing bis(crown)stilbene (*E*)-**1** (π-donor, D) and viologen analog (π-acceptor, A) in concentrations of up to 0.05 and 1 × 10^−3^ M, respectively, are well described with the complexation model that includes the following equilibria:

(1) $$A+D\overset{K_{1}}{↔}D\cdot A$$

(2) $$D\cdot A+D\overset{K_{2}}{↔}D\cdot A\cdot D$$

where *K*_1_/M^−1^ and *K*_2_/M^−1^ are the stability constants of bi- and termolecular complexes. We found that this reaction model is also applicable to the donor–acceptor system investigated in the present work (Scheme 3).

The major driving force of the reaction giving the pseudocyclic bimolecular D·A complex is the homoditopic (two-center) binding of ammonium groups to crown ether moieties through hydrogen bonds [18,23]. This binding is most efficient when the geometric parameters of the components exactly match. Otherwise, steric strain in the bimolecular complex promotes the formation of termolecular sandwich complexes in which the acceptor molecule is sandwiched between two donor molecules. The planar geometry of the conjugated moieties of the donor and acceptor molecules provides an additional moderate contribution to the stability of complexes due to the donor–acceptor and stacking interactions.

The main spectral characteristics and stability constants of the bi- and termolecular complexes of stilbene derivative (*E*)-**1** with diammonium acceptors (*E*)-**2** and (*Z*)-**2** are presented in Table 1.

### 2.3. ^1^H NMR Spectroscopy

**Figure 1:**
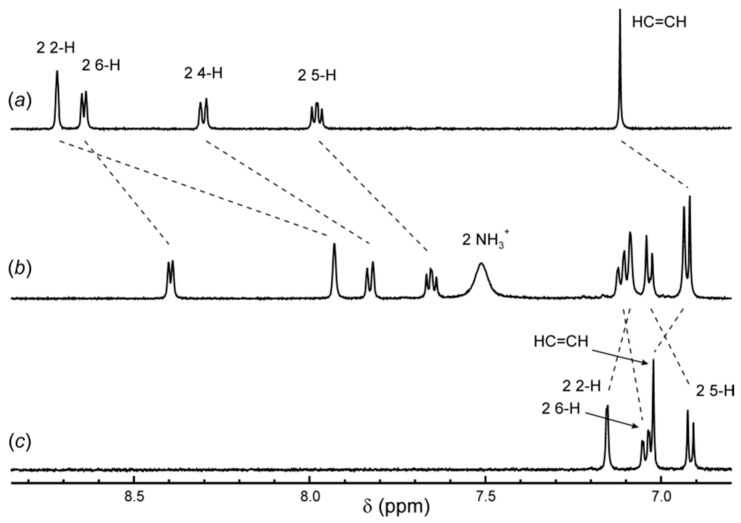
^1^H NMR spectra (aromatic proton region) of (**a**) (*Z*)-**2** (*C* = 1 × 10^−3^ M^−1^), (**c**) (*E*)-**1** (*C* = 1 × 10^−3^ M^−1^), and (**b**) their equimolar mixture, MeCN-*d*_3_, 25 °C.

**Figure 2:**
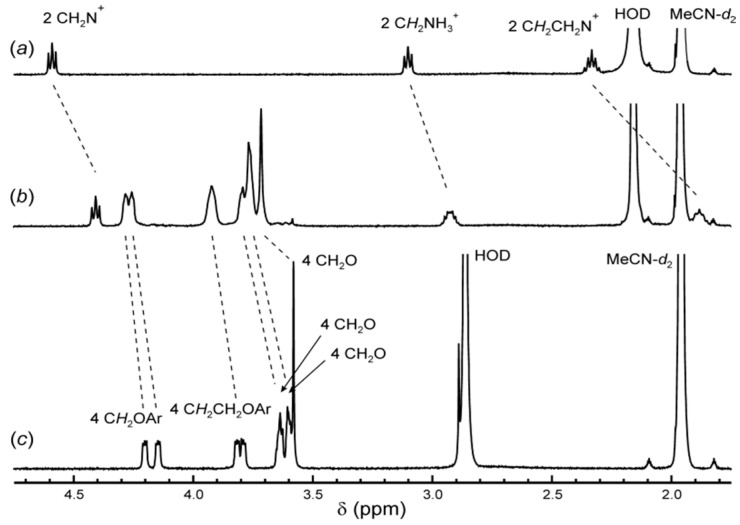
^1^H NMR spectra (aliphatic proton region) of (**a**) (*Z*)-**2** (*C* = 1 × 10^−3^ M^−1^), (**c**) (*E*)-**1** (*C* = 1 × 10^−3^ M^−1^), and (**b**) their equimolar mixture, MeCN-*d*_3_, 25 °C.

**Figure 3:**
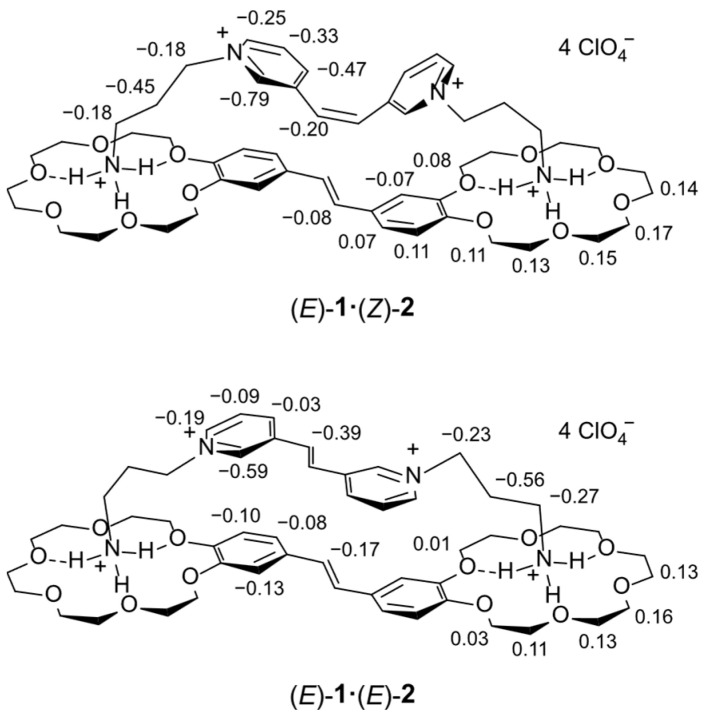
Chemical shift changes for hydrogens, Δ*δ*_H_ = *δ*_H_(complex D·A) − *δ*_H_ (free component), upon complexation of (*E*)-**1** with (*Z*)-**2** and (*E*)-**2**, MeCN-*d*_3_, 25 °C. Data for (*E*)-**1·**(*E*)-**2** were taken from Ref. [18].

When (*E*)-**1** is added to a solution containing an equimolar amount of (*Z*)-**2** in MeCN-*d*_3_, the positions of most signals in the ^1^H NMR spectra considerably change (Δ*δ*_H_, Figure 1 and Figure 2, see atom numbering in Scheme 2). This is due to the formation of pseudocyclic D**·**A complex in which the components are located one above the other (Scheme 3). Figure 3 presents the Δ*δ*_H_ values for an equimolar mixture of bis(crown)stilbene (*E*)-**1** with diammonium compound (*Z*)-**2**. For comparison, analogous data for a mixture of (*E*)-**1** and (*E*)-**2** is shown. The coordination via hydrogen bonding between the ammonium groups of acceptors and the 18-crown-6 ether moieties of (*E*)-**1** induces a downfield shift in signals of most CH_2_O groups (Δ*δ*_H_ of up to 0.17 ppm), which is due to the electron-withdrawing effect of the ammonium ion. In the D·A complexes, the central moieties of the components are located closely to each other, and most proton signals of both the donor and the acceptor are shifted upfield as a result of mutual shielding (Δ*δ*_H_ up to –0.79 ppm). As the content of (*E*)-**1** in the solution increases, the proton signals of the acceptor continue to shift upfield. This indicates switching from the bimolecular D**·**A complex to the termolecular D**·**A**·**D complex in which the acceptor molecule is sandwiched between two bis(crown)stilbene molecules (Scheme 3) and, hence, the hydrogen atoms of the acceptor are even more shielded.

The stability of the supramolecular complexes was quantitatively determined by ^1^H NMR titration in the (*E*)-**1**/(*Z*)-**2** system. The resulting dependences of Δ*δ*_H_ for the proton signals of the acceptor on the amount of (*E*)-**1** added correspond to the formation of 1:1 and 2:1 complexes (Equations (1) and (2)). Due to the very high stability of the bimolecular D·A complex exceeding the upper threshold of applicability of the ^1^H NMR titration method (*K*_1_ > 10^5^ M^−1^), the *K*_1_ value was determined by competitive ^1^H NMR titration using 1,10-diammoniodecane diperchlorate (**C10**) and the stability constant of complex (*E*)-**1·C10** determined previously by spectrophotometric titration (log*K*_1_ = 7.58 [25]). The log*K*_1_ and log*K*_2_ values obtained in this way are given in Table 1. The data for similar complexes involving (*E*)-**2** and (*E*)-**3** are given in the Table for comparison. As was to be expected, going from (*Z*)-**2** to (*E*)-**2**(**3**) leads to increasing *K*_1_ and *K*_2_ due to enhanced stacking interactions in the complexes formed by planar acceptor molecules. The stability of the termolecular complex formed by (*Z*)-**2** is very low, which is apparently indicative of the optimal geometric matching between the components in the preceding bimolecular complex (*E*)-**1·**(*Z*)-**2** for the homoditopic binding by hydrogen bonds.

### 2.4. X-Ray Crystallography

**Figure 4:**
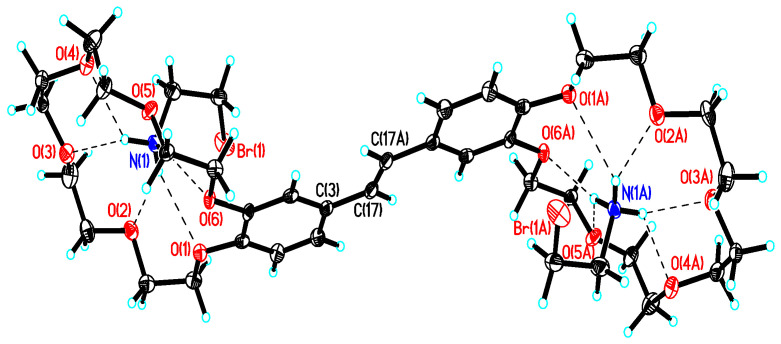
Structure of the main components in (*E*)-**1·**8C_2_H_7_Br_2_N. Thermal ellipsoids are drawn at the 50% probability level. Hydrogen bonds are shown by dashed lines. Symbols A are added to the atoms that belong to the symmetrically related sites.

The complex of stilbene (*E*)-**1** with 2-bromoethylammonium bromide was obtained in the crystalline state and studied by X-ray diffraction. The main components of the obtained structure (*E*)-**1·**8C_2_H_7_Br_2_N are shown in Figure 4; the crystal data and X-ray experiment details are summarized in Section 3.3.3.

Molecule (*E*)-**1** is located at the center of symmetry of the crystal, which coincides with the middle of the C(17)=C(17A) bond; therefore, the conjugated moiety has a perfectly planar conformation. The bond length distribution in the ethylene moiety is typical of stilbene derivatives: the bond lengths in the C(3)–C(17)=C(17A) moiety are 1.465(6) and 1.324(8) Å, respectively. This attests to a significant localization of the ethylene bond in (*E*)-**1**, as in the previously studied crystalline free (*E*)-**1** and its complexes with diammonium compounds [18,19,23,26,27,28].

The crown ether moieties of (*E*)-**1** coordinate two BrCH_2_CH_2_NH_3_^+^ ions located on different sides of the chromophore plane through the formation of six bifurcated hydrogen bonds by each ion. The N(1)H^…^O(1, …, 6) bond lengths vary in the 2.09–2.40 Å range, and the angles at the H atoms vary from 122° to 159°. The distance between the N(1) and N(1A) atoms is 14.18 Å. Three more independent 2-bromoethylammonium ions forming numerous hydrogen bonds with the bromide anions were found in (*E*)-**1·**8C_2_H_7_Br_2_N structure.

**Figure 5:**
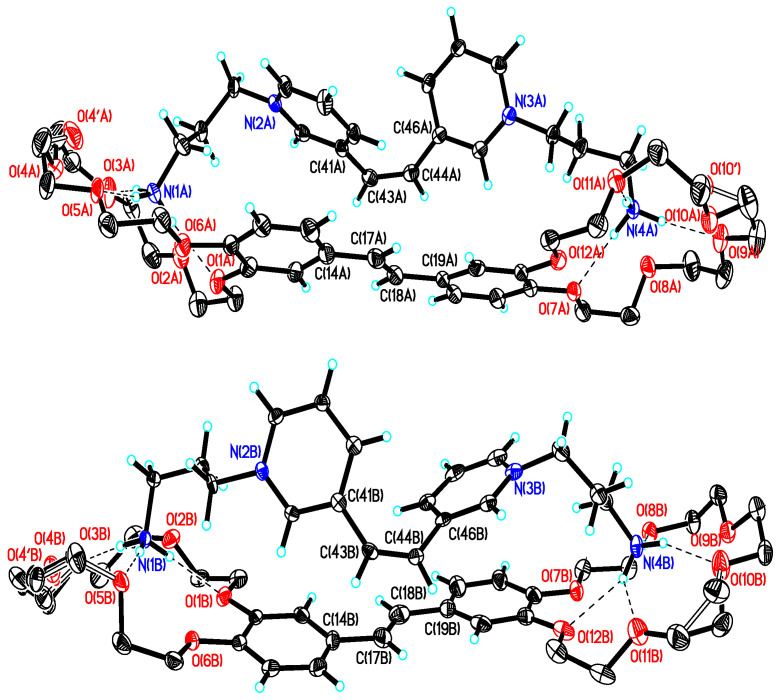
Structures of the main components in [(*E*)-**1·**(*Z*)-**2**]**·**0.15 MeCN**·**1.275 H_2_O (two independent complexes). Thermal ellipsoids are drawn at the 40% probability level. Minor components of the disorder are shown with hollow lines. Hydrogen bonds are drawn with dashed lines.

We were also able to obtain a supramolecular complex of (*E*)-**1** with (*Z*)-**2** as yellowish single crystals suitable for X-ray crystallography. The structures of the main components of [(*E*)-**1·**(*Z*)-**2**]**·**0.15 MeCN**·**1.275 H_2_O are shown in Figure 5; the crystal parameters and X-ray experiment details are given in Section 3.3.3.

The structure of [(*E*)-**1·**(*Z*)-**2**]**·**0.15 MeCN**·**1.275 H_2_O is fairly loose; the voids between the main components in the crystal lattice are occupied by the perchlorate anions (half of them are disordered) and MeCN and water molecules of solvation. It is evident that these small species do not perfectly fit into the voids in the lattice in the shape and size, which allows the ClO_4_^−^ ions to either execute various vibrations or be generally disordered over two close positions, the MeCN(1S) and water H_2_O(2W, …, 8W) molecules have partial site occupancy, and four parts of the macrocycles in stilbene molecules (*E*)-**1** show conformational disorder. All the above features are usual for the crystalline crown ether-based supramolecular structures [19,23,29]. They markedly decrease the reflectivity of the crystal and, hence, deteriorate the accuracy of the X-ray diffraction experiment.

Two independent pseudocyclic bimolecular D·A complexes (see Scheme 3), differing somewhat in the conformations of components, were found in the structure of [(*E*)-**1·**(*Z*)-**2**]**·**0.15 MeCN**·**1.275 H_2_O. This geometric scatter points to the non-rigidity of this system. All the (CH_2_)_3_NH_3_^+^ groups in these complexes occur in the *trans*,*gauche*- or *gauche*,*trans*-conformations. The distances between the nitrogen atoms of the ammonium groups, N(1A)…N(4A) and N(1B)…N(4B), are 14.46 and 14.61 Å. The stilbene moieties in both independent molecules (*E*)-**1** in the complexes are nearly planar: the dihedral angles between the planes of the benzene rings are only 8.1° and 9.6°. Conversely, the conjugated moieties of both independent molecules of the acceptor in the *Z*-isomer are markedly twisted: the dihedral angles between the planes of the C_5_H_4_N(2A)/C_5_H_4_N(3A) and C_5_H_4_N(2B)/C_5_H_4_N(3B) pyridine rings are 49.0° and 60.0°, respectively. The non-planar structure of the acceptor precludes considerable stacking interactions in (*E*)-**1·**(*Z*)-**2**.

The bond length distributions in the ethylene moieties of (*E*)-**1** and (*Z*)-**2** are similar: in the C(14A,B)–C(17A,B)=C(18A,B)–C(19A,B) and C(41A,B)–C(43A,B)=C(44A,B)–C(46A,B) bridges, the lengths of these bonds are, on average, 1.492(6), 1.294(6), 1.497(6) Å and 1.478(6), 1.325(6), 1.475(6) Å. The NH_3_^+^ groups of (*Z*)-**2** form mainly directed hydrogen bonds with the oxygen atoms of the crown ether moieties of (*E*)-**1**. The hydrogen bond parameters are as follows: the NH^…^O distances vary in the range of 1.85–2.23 Å and the angles at the H atoms vary from 125° to 175°. These values correspond to medium-strength hydrogen bonds.

In the crystal, the independent complexes (*E*)-**1·**(*Z*)-**2** form separate slanted, very loose stacks of the type (…A**·**D···D**·**A…A**·**D···D**·**A…)*_n_* (Figure S6 in Supplementary Materials). Stacking interactions are possible in these stacks only between the conjugated moieties of neighboring donor molecules arranged in parallel planes at distances of ~3.40–3.45 Å, but their mutual projections are small. The conjugated moieties of the neighboring acceptor molecules are virtually not projected onto each other.

### 2.5. Intrasupramolecular Photo-Driven Electron Transfer in Complexes (E)-1·(Z)-2, (E)-1·(E)-2

#### 2.5.1. Steady-State Spectroscopy

The spectral characteristics of complexes 1(D):1(A) and the free components are summarized in Table 2. Excitation of the donor component in (*E*)-**1·**(*Z*)-**2** and (*E*)-**1·**(*E*)-**2** results in a much lower fluorescence quantum yield (*ϕ*_f_) than that observed for free stilbene (*E*)-**1**. Due to the overlap of the absorption spectra of the donor and acceptors, it is impossible to selectively excite only the acceptor components of the complexes. A different nature of fluorescence was noted for pseudocyclic complex (*E*)-**1·C10** devoid of the aromatic acceptor moiety. Due to the absence of photo-driven charge transfer (D → A), the fluorescence quantum yield of this compound is fairly high. The fact that the *ϕ*_f_ value was two orders of magnitude higher for complex (*E*)-**1·**(*E*)-**2** than for isomeric complex (*E*)-**1·**(*E*)-**3** (*ϕ*_f_ < 10^−4^ [16]) suggests much less effective photo-driven electron transfer in the former case.

The results of steady-state spectroscopy suggest that fluorescence in free stilbene and in complex (*E*)-**1·C10** occurs from the LE states with electronic configurations similar to those for the ground states. Conversely, the fluorescent states of complexes (*E*)-**1·**(*Z*)-**2** and (*E*)-**1·**(*E*)-**2** are presumably dynamically formed CT states that are more polarized than the primarily excited Frank–Condon states. The increased solvation of the partially separated charges decreases the energy of (*E*)-**1·**(*Z*)-**2** and (*E*)-**1·**(*E*)-**2**, which increases the Stokes shift (Table 2). The sample fluorescence at low temperatures was measured to find out whether there is an energy barrier for the photo-driven charge transfer reaction. The fluorescence spectra of stilbene and complexes (*E*)-**1·**(*Z*)-**2**, (*E*)-**1·**(*E*)-**2**, and (*E*)-**1·C10** in vitrifying *n*-PrCN at 293 and 77 K showed the following behavior. On going from a liquid solution to the solid low-temperature matrix, the blue shift in the fluorescence maxima of stilbene and complex (*E*)-**1·C10** is ~4 nm, while those for complexes (*E*)-**1·**(*Z*)-**2** and (*E*)-**1·**(*E*)-**2** are 1.5 and 2 nm. The areas under fluorescence spectra of the samples in the 293–77 K range increase by factors of only 2.12 ((*E*)-**1**), 1.0 ((*E*)-**1·C10**), 2.3 ((*E*)-**1·**(*Z*)-**2**), and 1.05 ((*E*)-**1·**(*E*)-**2**). This moderate enhancement of fluorescence implies that the intramolecular photo-driven charge transfer is retained at 77 K and that there is no significant barrier between the LE and CT states of complexes (*E*)-**1·**(*Z*)-**2** and (*E*)-**1·**(*E*)-**2**. Similar conclusions were drawn in a study of the intrasupramolecular photo-driven electron transfer in related stilbene–viologen complexes [17,20].

#### 2.5.2. Transient Absorption

In order to determine the pathway of relaxation of the singlet excited states of acceptors and their complexes, we carried out time-resolved measurements of the S_1_–S*_n_* absorption spectra (TA spectra). The spectral evolution of free donor (*E*)-**1** was studied previously [16,21,22] and is not considered here.

**Figure 6:**
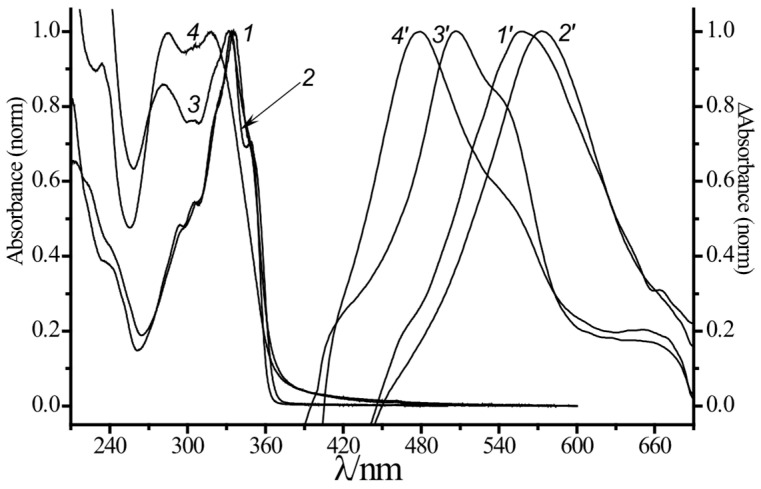
Absorption spectra of (*E*)-**1** (1) and complexes (*E*)-**1·C10** (2), (*E*)-**1·**(*Z*)-**2** (3), and (*E*)-**1·**(*E*)-**2** (4), (*C*_D_/*C*_A_ = 1/1) in MeCN (left). Difference absorption spectra of (*E*)-**1** (1′), (*E*)-**1·C10** (2′), (*C*_D_/*C***_C10_** = 1/4, *λ*_exc_ = 330 nm), (*E*)-**1·**(*Z*)-**2** (3′), and (*E*)-**1·**(*E*)-**2** (4′), (*C*_D_/*C*_A_ = 1/4, *λ*_exc_ = 350 nm) in the saturation region (0.35–0.6 ps) in MeCN (right).

The TA spectra of acceptor (*E*)-**2** show fast (in ~400 fs) buildup of a broad band at 383 nm, which is blue-shifted during saturation by 17 nm within 10 ps. This is followed by a monoexponential decay of this band in the ns range without any shift. The spectral behavior of acceptor (*Z*)-2 is generally similar, including a slower buildup of a broad noisy (without a clear maximum) TA band, which, after reaching saturation, virtually does not decay up to 500 ps. Owing to the blue shift in the absorption bands of the acceptors (*Z*)-**2** and (*E*)-**2** (Table 2, Figure 6) relative to the spectrum of the donor, we are able to selectively excite the donor in the complexes.

**Figure 7:**
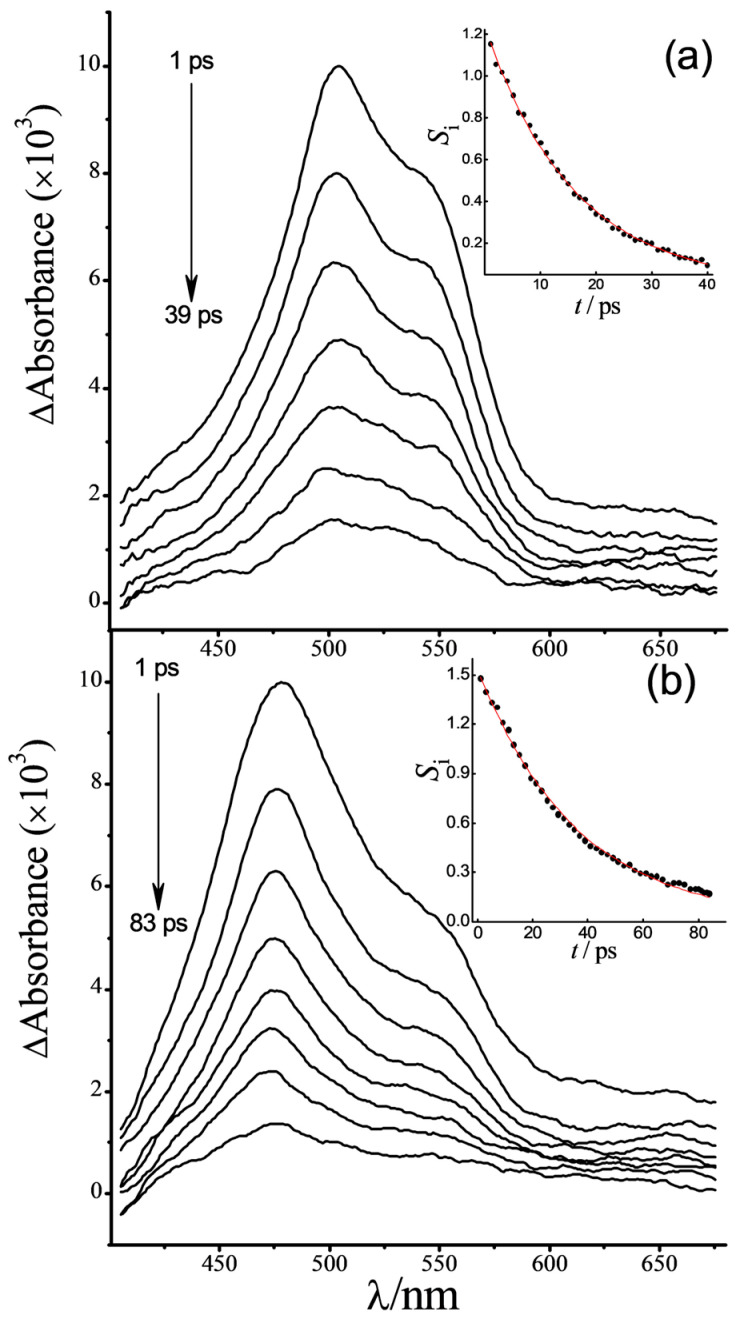
Difference absorption of and (*E*)-**1·**(*Z*)-**2** (spline) within 1–39 ps (**a**) and (*E*)-**1·**(*E*)-**2** (spline) within 1–83 ps (**b**) after excitation in MeCN with a 30-fs pulse at 350 nm. Insets: time plot of the areas under the transient absorption spectra of (*E*)-**1·**(*Z*)-**2** (**a**) and (*E*)-**1·**(*E*)-**2** (**b**). The smooth curves are from fitting to a monoexponential: y = 1.233 × exp(–t/16) (**a**), y = 1.529 × exp(–t/36) (**b**).

The evolution of the TA spectra of complexes (*E*)-**1·**(*Z*)-**2** and (*E*)-**1·**(*E*)-**2** in MeCN is as follows. During the first 0.69 and 1.19 ps after a 30 fs excitation at 350 nm, buildup of the band at 504 or 477 nm for (*E*)-**1·**(*Z*)-**2** or (*E*)-**1·**(*E*)-**2**, respectively, is observed. Then this band decays without any shift with characteristic time of 16 or 36 ps, respectively; this is consistent with the approximately twofold difference between the *ϕ*_f_ values of these complexes (Table 2). In both cases, the overall spectral kinetics is well described by a biexponent (Table 3). The steady-state and S_1_–S*_n_* absorption spectra of the donor and the complexes are shown in Figure 6. The evolution of the TA spectra of (*E*)-**1·**(*Z*)-**2** and (*E*)-**1·**(*E*)-**2** in the ps range, together with the decay kinetics, is shown in Figure 7. The evolution of the spectra of complex (*E*)-**1·C10** follows a different pattern. The buildup of a single TA band with a monotonic ~10 nm blue shift takes place in 350 fs, with the absorption intensity being retained for up to 46 ps. Then this band decays without any shift with a characteristic time of 0.69 ns. Since the pseudocyclic complex (*E*)-**1·C10** has no aromatic acceptor moiety, the intrasupramolecular D-A electron transfer does not take place in this case. Meanwhile, the ammonium ions coordinated to the crown ether moieties increase the polarity of the complex. This accounts for the 12 nm red shift in the TA band of (*E*)-**1·C10** relative to that of (*E*)-**1** (Figure 6). The observed ultrafast buildup of the 573 nm TA band of complex (*E*)-**1·C10** is probably due to the vibrational relaxation into the fluorescent LE state. It does not noticeably change up to 46 ps and then it is deactivated to the ground state in the ns range.

Previously, evolution of the TA spectra of isomeric complex (*E*)-**1·**(*E*)-**3** (see Scheme 1) upon excitation at 308 nm was investigated [16]. The evolution follows a triexponential kinetics with characteristic intensity buildup times of 150 and 295 fs and decay times of 536 fs (back ET). As has already been noted for complexes (*E*)-**1·**(*Z*)-**2** and (*E*)-**1·**(*E*)-**2** and similar excitation conditions, the kinetics is biexponential. It follows from Figure 6 that the long-wavelength absorption band in the ground state of these complexes is blue-shifted relative to that of free donor by 4 and 18 nm, respectively. The shorter-wavelength peaks at 281 and 285 nm correspond to the absorption of acceptors in the complexes. The analogous shifts in the TA spectra of complexes (*E*)-**1·**(*Z*)-**2** and (*E*)-**1·**(*E*)-**2** proved to be much higher: 57 and 84 nm. This indicates that the TA spectra of complexes (*E*)-**1·**(*Z*)-**2** and (*E*)-**1·**(*E*)-**2** shown in Figure 6 reflect the absorption of ultrafast-formed CT states with charges partially transferred to the acceptors. In all probability, the *τ*_1_ values (Table 3) characterize here the direct intrasupramolecular D-A electron transfer together with the vibrational relaxation of the CT state. Due to the ultrafast charge transfer from the Franck–Condon excited state, the relaxed LE state of the complexes is probably not populated. It is important that *τ*_2_ values of complexes (*E*)-**1·**(*Z*)-**2** and (*E*)-**1·**(*E*)-**2** are much greater than *τ*_3_ for isomeric complex (*E*)-**1·**(*E*)-**3** (Table 3). This suggests that the back diabatic electron transfer in them is largely retarded. The rate constant for the back electron transfer for (*E*)-**1·**(*E*)-**2** is ~1.5% relative to that for the isomeric complex (*E*)-**1·**(*E*)-**3**.

### 2.6. Quantum Chemical Calculations

**Figure 8:**
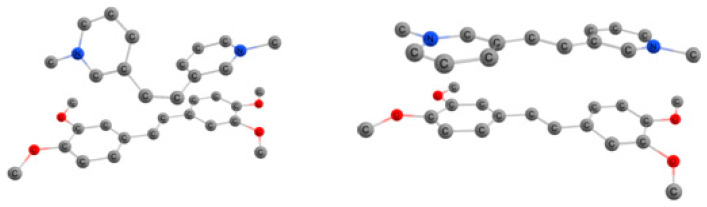
Examples of model complexes for (*E*)-**1·**(*Z*)-**2** and (*E*)-**1·**(*E*)-**2** in which the peripheral aliphatic moieties have been removed.

The geometric parameters of complexes in the S_0_ and S_1_ states were determined. The optimized structures of the complexes in the S_0_ and S_1_ states are shown in Figures S7 and S8 (Supplementary Materials). For complexes (*E*)-**1·**(*E*)-**2** and (*E*)-**1·**(*E*)-**3**, the conformations with flattened aromatic moieties of the donor and the acceptor are energetically favorable (Table 4). The distances between atoms of the donor and the acceptor atoms located most closely vary from 3.4 to 4.2 Å. In complex (*E*)-**1·**(*E*)-**2**, the aromatic moieties of the donor and the acceptor are nearly arranged in a stack in which effective stacking interactions occur (Figure 8). In isomeric (*E*)-**1·**(*E*)-**3**, these moieties are somewhat shifted relative to each other in parallel planes, which increases the distance between the donor and acceptor centers of mass and, hence, weakens the stacking interactions. The acceptor components of (*E*)-**1·**(*Z*)-**2** and (*E*)-**1·**(*Z*)-**3** are markedly twisted in both S_0_ and S_1_ states. Therefore, stacking interactions in complexes with *Z*-isomers are unlikely, which is in good agreement with the X-ray diffraction data for complex (*E*)-**1·**(*Z*)-**2** (see above).

**Figure 9:**
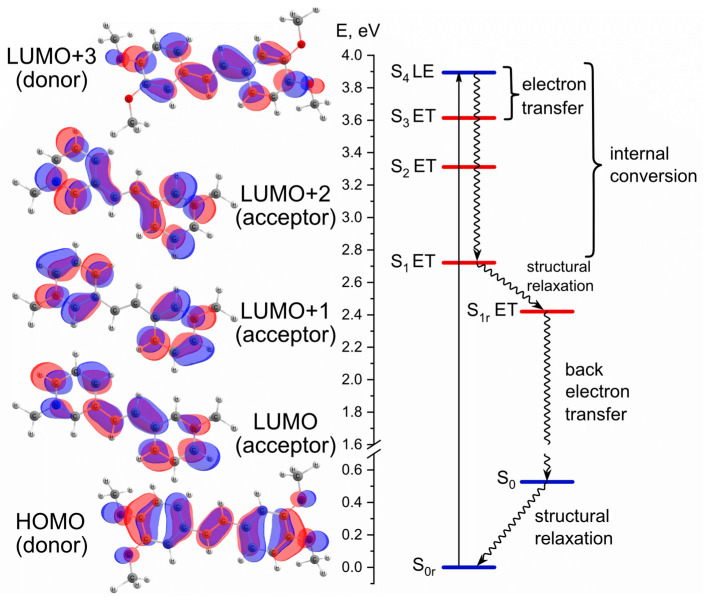
Orbitals involved in the formation of excited states (S_1_–S_4_) for the complex modeling complex (*E*)-**1·**(*E*)-**2** and the putative photorelaxation pathways for this complex.

In the simulated absorption spectra, one can distinguish three weak long-wavelength bands corresponding to donor–acceptor transfers (Table S1 in Supplementary Materials). In complexes (*E*)-**1·**(*Z*)-**2** and (*E*)-**1·**(*E*)-**2**, they occur from HOMO of the donor to LUMO, LUMO+1, and LUMO+2 of the acceptor. Two intense bands at 317 and 300 nm correspond to the transfers localized on the donor (HOMO-LUMO+3) or on the acceptor (HOMO-4-LUMO) (Figure 9).

Thus, the following photorelaxation pathway can be proposed for complex (*E*)-**1·**(*E*)-**2**. 1. The excitation of the main long-wavelength absorption band mainly results in population of the LE state of the donor, since the oscillator strength of the donor–acceptor transfers is low. 2. The LE state undergoes an ultrafast electron transfer from LUMO+3 to LUMO+2 (S_4_→S_3_), which is facilitated by the relatively low estimated value of the energy gap between the states (~0.3 eV). 3. The resulting ET state is expected to undergo even faster internal conversion S_3_→S_2_→S_1_, consisting in the electron transfer within the acceptor (from LUMO+2 to LUMO). 4. In the S_1_ state, which has a rather long lifetime, several competing processes can take place, such as structural relaxation, internal conversion, and intersystem crossing. In this case, the back electron transfer from the acceptor LUMO to the donor HOMO is the main channel of non-radiative deactivation to the ground state. The 67-fold higher estimated rate constant of the back electron transfer in complex (*E*)-**1·**(*E*)-**3** (1.87 × 10^12^ s^−1^) compared to that of (*E*)-**1·**(*E*)-**2** (2.78 × 10^10^ s^−1^) is difficult to explain on the basis of the data of Table 4. In the case of complex (*E*)-**1·**(*Z*)-**2** closely related to the latter, this rate constant is only ~2.2 times higher (6.25 × 10^10^ s^−1^).

## 3. Materials and Methods

### 3.1. Materials

MeCN (Panreac, HPLC gradient grade, water content of 0.02%, and Cryochrom, special purity grade, water content *<* 0.03%) was used to prepare solutions. *n*-PrCN (Merck, spectral grade) was dried by distillation from CaH_2_. Bis(18-crown-6)stilbene ((*E*)-**1**) [26], 4,4′-(*E*)-ethene-1,2-diylbis [1-(3-ammoniopropyl)pyridinium] tetraperchlorate ((*E*)-**3**), (*Z*)-di(3-pyridyl)ethylene ((*Z*)-**4**) [18], 1,10-diammoniodecane diperchlorate (**C10**) [29], and 1,12-diammoniododecane diperchlorate (**C12**) [18] were synthesized by known procedures. 2-Bromoethylammonium bromide, 3-bromopropylammonium bromide, and HClO_4_ (70%, aq.) (Sigma–Aldrich, St. Louis, MO, USA) were used as received. Compounds (*E*)-**2**, (*Z*)-**2**, (*E*)-**3**, **C10**, **C12**, and their complexes containing perchlorate anions are nonexplosive.

### 3.2. Synthesis of 3,3′-(Z)-Ethene-1,2-diylbis[1-(3-ammoniopropyl)pyridinium] Tetraperchlorate ((Z)-2)

A solution of a mixture of compound (*Z*)-**4** (143 mg, 0.79 mmol) and 3-bromopropylammonium bromide (1.03 g, 4.71 mmol) in dried MeCN (14 mL) was heated at 80 °C with stirring for 32 h. The reaction mixture was cooled to 5 °C, and a precipitate thus formed was filtered, washed with abs. EtOH (3 × 10 mL), and dried in air to give the corresponding tetrabromide salt of the acceptor (396 mg, yield 81%) as a yellowish powder. The tetrabromide salt (206 mg, 0.33 mmol) was dissolved in a mixture of EtOH (10 mL) and a minimum amount of water at heating, and then HClO_4_ (70%, aq.) (0.26 mL, 2.58 mmol) was added to the solution. The resulting solution was cooled to 5 °C and a precipitate thus formed was filtered, washed with abs. EtOH (2 × 5 mL), and dried in air to give compound (*Z*)-**2** (209 mg, yield 91%) as a white powder, mp 236–237 °C dec. Calcd for C_18_H_28_Cl_4_N_4_O_16_ (698.24): C, 30.96; H, 4.04; N, 8.02; found: C, 30.76; H, 4.05; N, 7.99%. ^1^H NMR (DMSO-*d*_6_, *J*/Hz, 30 °C) *δ* 2.17 (m, 4 H, 2 C*H*_2_CH_2_N), 2.86 (m, 4 H, 2 C*H*_2_NH_3_), 4.60 (t, 4 H, *J* = 7.3, 2 CH_2_N), 7.13 (s, 2 H, HC=CH), 7.74 (br s, 6 H, 2 NH_3_), 8.04 (dd, 2 H, *J* = 8.2, *J* = 6.1, 2 5-H), 8.40 (d, 2 H, *J* = 8.2, 2 4-H), 8.95 (d, 2 H, *J* = 6.1, 2 6-H), 9.09 (s, 2 H, 2 2-H) ppm. ^13^C NMR (DMSO-*d*_6_, 26 °C) *δ* 28.51 (2 *C*H_2_CH_2_NH_3_), 35.72 (2 CH_2_NH_3_), 58.04 (2 CH_2_N), 128.19 (2 5-C), 128.71 (HC=CH), 135.25 (2 3-C), 143.84 (2 6-C), 144.40 (2 4-C), 144.79 (2 2-C) ppm. See Figures S1 and S2 in Supplementary Materials. IR (Nujol), *ν*/cm^−1^: 3222 (N^+^–H).

#### 3.2.1. Synthesis of Complex (E)-1·(Z)-2

A solution of a mixture of compounds (*E*)-**1** (16.9 mg, 26.0 μmol) and (*Z*)-**2** (18.1 mg, 25.9 μmol) in MeCN (4 mL) was slowly saturated with benzene vapor at room temperature for 2 weeks in the dark. The precipitate thus formed was decanted and dried in air to give complex (*E*)-**1·**(*Z*)-**2** (30.8 mg, 88% yield) as yellowish crystals, mp > 265 °C dec. Calcd for C_34_H_48_O_12_·C_18_H_28_Cl_4_N_4_O_16_ (1346.98): C, 46.37; H, 5.69; N, 4.16; found: C, 46.31; H, 5.67; N, 4.09%. IR (Nujol), *ν*/cm^−1^: 3180 (N^+^–H). The formal 1:1 stoichiometry for this complex was confirmed by ^1^H NMR spectroscopy data (in DMSO-*d*_6_, see Figure S3 in Supplementary Materials).

#### 3.2.2. 3,3′-(E)-Ethene-1,2-diylbis[1-(3-ammoniopropyl)pyridinium] Tetraperchlorate 

((E)-2) was prepared according to a described procedure [18]. ^1^H NMR (DMSO-d_6_, J/Hz, 25 °C) δ 2.23 (m, 4 H, 2 CH_2_CH_2_N), 2.89 (m, 4 H, 2 CH_2_NH_3_), 4.68 (t, 4 H, J = 7.1, 2 CH_2_N), 7.72 (s, 2 H, HC=CH), 7.75 (br s, 6 H, 2 NH_3_), 8.29 (dd, 2 H, J = 8.4, J = 6.2, 2 5-H), 8.83 (d, 2 H, J = 8.4, 2 4-H), 9.02 (d, 2 H, J = 6.2, 2 6-H), 9.27 (s, 2 H, 2 2-H) ppm. ^13^C NMR (DMSO-d_6_, 25 °C) δ 28.44 (2 CH_2_CH_2_NH_3_), 35.73 (2 CH_2_NH_3_), 58.32 (2 CH_2_N), 128.23 (HC=CH), 128.44 (2 5-C), 135.90 (2 3-C), 142.20 (2 4-C), 143.34 (2 2-C), 144.04 (2 6-C) ppm. See Figures S4 and S5 in Supplementary Materials.

### 3.3. Methods

Melting points (uncorrected) were measured in capillaries on a Mel-Temp II apparatus. Elemental analyses were carried out at the Microanalytical Laboratory of the A. N. Nesmeyanov Institute of Organoelement Compounds of the Russian Academy of Sciences (Moscow, Russia). The samples for elemental analyses were dried in vacuo at 80 °C. ^1^H and ^13^C NMR spectra were recorded on a Bruker DRX500 instrument (Billerica, MA, USA) (500.13 and 125.76 MHz, respectively) in DMSO-*d*_6_ and MeCN-*d*_3_ at 25–30 °C using the signal from the solvent as the internal standard (*δ*_H_ 2.50 and 1.96, respectively; *δ*_C_ 39.43 for DMSO-*d*_6_). Chemical shifts were measured with an accuracy of 0.01 ppm and spin–spin coupling constants were determined with an accuracy of 0.1 Hz. ^1^H–^13^C correlation spectra (HSQC) were used to assign the hydrogen and carbon signals (see Scheme 2 for atom numbering). IR spectra were registered on a Bruker ISF-113V spectrometer (Billerica, MA, USA) n Nujol between KBr plates. Fluorescence spectra at 77 K were recorded using a quartz Dewar microvessel in the *n*-PrCN matrix.

#### 3.3.1. Absorption and Fluorescence Spectroscopy

Steady state absorption spectra were recorded on a Shimadzu-3100 (Kyoto, Japan) and a Specord-250 Plus spectrophotometers (Analytik Jena, Germany). Steady state fluorescence spectra were measured on a Perkin-Elmer LS-55 (Beaconsfield, UK) and an Agilent Cary Eclipse spectrofluorimeter (Santa Clara, CA, USA). Fluorescence quantum yields were determined relative to quinine sulfate in 1.0 *N* H_2_SO_4_ (*ϕ*_f_ = 0.546 [30]) with equal absorbances at the excitation wavelength. The measurements were carried out with freshly prepared aerated solutions.

#### 3.3.2. Titrations

The direct and competitive ^1^H NMR titration experiments were carried out in MeCN-*d*_3_ solutions at 25 °C. In the direct titration, the stability constant for the termolecular D**·**A**·**D complex (*K*_2_, M^−1^) was determined by analyzing the shifts in the hydrogen signals of the acceptor (*Z*)-**2** (Δ*δ*_H_, in the region *C*_D_ > *C*_A_) as a function of the concentration of the added donor (*E*)-**1** [23]. The total concentration of (*Z*)-**2** was maintained constant at ~1 × 10^−3^ M^−1^, and the total concentration of (*E*)-**1** varied from 0 to ~1.1 × 10^−2^ M^−1^. In the competitive titration, the stability constants for the bimolecular D**·**A complexes (*K*_1_, M^−1^) were measured as follows: the total concentrations of (*E*)-**1** and the acceptor were maintained constant at ~1.2 × 10^−3^ M^−1^ and ~1 × 10^−3^ M^−1^, respectively, and the total concentration of the competing reactant, 1,10-diammoniodecane diperchlorate (**C10**), varied from 0 to ~1.2 × 10^−2^ M^−1^. The titration data were treated using the HYPNMR program [31]. The competitive spectrofluorimetric titration was carried out in MeCN at 22 °C. The stability constant for the bimolecular D**·**A complex (*K*_1_, M^−1^) was determined as follows [23]: the total concentrations of (*E*)-**1** and (*Z*)-**2** were maintained constant at ~1.7 × 10^−5^ M^−1^ and ~3.0 × 10^−5^ M^−1^, respectively, and the total concentration of the competing reactant, 1,12-diammoniododecane diperchlorate (**C12**), varied from 0 to 1.2 × 10^−3^ M^−1^. Log*K*_1_ was calculated with Equation (3):

log*K*_1_ = log*K*_c_ − log*K*_s_ (3)

where *K*_1_ is the stability constant, while *K*_c_ is the formation constant for complex (*E*)-**1**·**C12** in reaction (4) (log*K*_c_ = 8.59 [23]) and *K*_s_ is the substitution constant of complex (*E*)-**1·**(*Z*)-**2** with acceptor **C12** in reaction (5).

(4) $$(E)\text{-}1+C12\;\overset{K_{C}}{↔}(E)\text{-}1\cdot C12$$

(5) $$(E)\text{-}1\text{·}(Z)\text{-}2\;+C12\;\overset{K_{S}}{↔}1\text{·}C12+(Z)\text{-}2$$

The value of log*K*_s_ = 0.11 was obtained by the analysis of fluorescence spectra for the system: (*E*)-**1·**(*Z*)-**2**/MeCN/**C12** in reaction (5). The initial 4-fold excess of acceptor (*Z*)-**2** was taken into consideration. The substitution constant was calculated with Equation (6):

(6) $$\frac{c^{(E)-1C12}}{c_{\max}^{(E)-1C12}}=\frac{1}{2c_{0}^{DA}}\left(-(c_{0}^{A}+K_{S}[C12])+\sqrt{{\left(c_{0}^{A}+K_{S}[C12]\right)}^{2}+4K_{S}c_{0}^{DA}[C12]}\right)$$

where *c***^(*E*)−1C12^**/*c*_max_**^(*E*)−1C12^** represents the ratio of current to maximum concentrations of (*E*)-**1**·**C12**, attained at the excess of **C12**; *c*_0_***^A^*** and *c*_0_***^DA^*** represent the initial concentrations of (*Z*)-**2** and (*E*)-**1·**(*Z*)-**2**, respectively. The total concentration of the competing reactant (**C12**), varied from 0 to ~1.0 × 10^−3^ M^−1^. Since (*E*)-**1·**(*Z*)-**2** exhibits a very weak fluorescence (*ϕ*_f_ = 0.006), we took the ratio under the fluorescence spectra of (*E*)-**1·**(*Z*)-**2**/MeCN/**C12** as *c***^(*E*)−1C12^**/*c*_max_**^(*E*)−1C12^**.

#### 3.3.3. X-Ray Crystallography

Single crystals of complexes (*E*)-**1·**8C_2_H_7_Br_2_N and [(*E*)-**1·**(*Z*)-**2**]**·**0.15 MeCN**·**1.275 H_2_O were grown as follows: a mixture of (*E*)-**1** with 2-bromoethylammonium bromide or compound (*Z*)-**2** was dissolved in MeCN, then the solution was slowly saturated with benzene by the vapor diffusion method at ambient temperature in the dark.

A selected single crystal was coated with perfluorinated oil and mounted on a Bruker SMART-CCD diffractometer (Billerica, MA, USA) [graphite monochromatized Mo-K_α_ radiation (*λ* = 0.71073 Å), *ω* scan mode] under a stream of cold nitrogen to measure crystallographic parameters and intensities of experimental reflections. Data reduction was performed using the SAINT program [32]. The structures were solved by direct methods and refined by least squares against *F*^2^ with anisotropic thermal parameters for all non-hydrogen atoms using the SHELXL 2014/7 software [33]. The hydrogen atoms were fixed at calculated positions at carbon and nitrogen atoms and then refined using a riding model. The hydrogen atoms of the water molecules of solvation in [(*E*)-**1·**(*Z*)-**2**]**·**0.15 MeCN**·**1.275 H_2_O were not located.

In the structure of (*E*)-**1·**8C_2_H_7_Br_2_N, the stilbene molecule is located at the center of symmetry of the unit cell. The Br(4) atom in the Br(4)CH_2_CH_2_N(4)H_3_^+^ ion is disordered over two positions with the occupancy ratio of 0.96:0.04. The SADI and ISOR commands were applied to constrain the geometry of this ion and anisotropic thermal parameters of the Br(4′) atoms.

In the structure of [(*E*)-**1·**(*Z*)-**2**]**·**0.15 MeCN**·**1.275 H_2_O, four of the eight independent perchlorate anions are strongly disordered over two positions each, with the occupancy ratios being 0.70:0.30, 0.73:0.27, 0.66:0.34, and 0.72:0.28. Four sites of the crown ether moieties of the stilbene molecules are disordered over two positions each, with the occupancy ratios being 0.73:0.27, 0.88:0.12, 0.56:0.44, and 0.81:0.19. The acetonitrile molecule of solvation has an occupancy of 0.30. The water molecules of solvation H_2_O(2W, …, 8W) have occupancies in the range from 0.15 to 0.30. This strong disorder reduces the accuracy of the X-ray diffraction experiment. The DFIX, SADI, and ISOR commands were used in the refinement.

The crystal data and structure refinement details are given in Table 5. The crystallographic data for (*E*)-**1·**8C_2_H_7_Br_2_N and [(*E*)-**1·**(*Z*)-**2**]**·**0.15 MeCN**·**1.275 H_2_O were deposited with the Cambridge Crystallographic Data Centre under numbers CCDC 2451335 and 2451336, respectively.

#### 3.3.4. Femtosecond Transient Absorption Spectroscopy

Femtosecond transient absorption (TA) spectra were measured at 22 °C on a pump and light supercontinuum probe setup with a 3.33 fs delay step and a 0.5 nm wavelength step. The spectra were corrected. The details of measurements of the transient spectra were described in our prior paper [34]. The characteristic times (*τ*) were calculated using the areas under single absorption bands in the appropriate time range. Since the experimental flash function convolution (FWHM = 30 fs) with monoexponential decay (*τ* = 50–80 fs) changes the *τ* value by only 1–2%, the experimental transient absorption kinetics corresponding to faster photo-driven electron transfer were processed without deconvolution. The average measurement error was ~5%. All measurements were performed in a darkroom under red light to prevent *E*–*Z* isomerization. In order to avoid excitation of free stilbene at 350 nm, the TA spectra of complexes (*E*)-**1·**(*Z*)-**2** and (*E*)-**1·**(*E*)-**2** were recorded for solutions containing a 4-fold excess of (*Z*)-**2** or (*E*)-**2**. The absorbance of the acceptor moiety in (*E*)-**1·**(*Z*)-**2** and (*E*)-**1·**(*E*)-**2** at 350 nm was negligible. The presence of a small amount of unstable complexes (*E*)-**1·**(*Z*)-**2·**(*E*)-**1** and (*E*)-**1·**(*E*)-**2·**(*E*)-**1** can be neglected. In view of the overlap with the ππ* absorption band of stilbene and the very low extinction of CT bands, fs-excitation of complexes at these bands was not performed.

#### 3.3.5. Quantum Chemical Calculations

Quantum chemical calculations were performed using the GAMESS (US) program package [35]. The ground-state geometry of the compounds was optimized by means of DFT; the excited state geometry was optimized by means of TDDFT (BHHLYP functional with the 6–31G(d,p) basis set). Absorption and emission spectra were calculated for model complexes by means of TDDFT (CAM-B3LYP ** functional with the def2-SVPD basis set). In all cases, the environmental effects were included via the Solvation Model Density continuum model [36]. The TDDFT method using the B3LYP and PBE functionals with low Hartree–Fock exchange usually adequately describes local transfers but may result in underestimated energies for donor–acceptor transfers. Here we used the TDDFT method with the CAM-B3LYP range-separated hybrid functional in which the Hartree–Fock exchange value depends on the distance. The spectral parameters were calculated for model complexes derived from the optimized structures of the complexes studied here by replacing some of the aliphatic groups of the donor and the acceptor with hydrogen atoms.

## 4. Conclusions

Bis(crown)stilbene (*E*)-**1** and diammoniopropyl di(3-pyridyl)ethylenes (*Z*)-**2** and (*E*)-**2** form highly stable pseudocyclic D**·**A complexes and very weak sandwich D**·**A**·**D complexes in MeCN due to numerous hydrogen bonds between the crown ether moieties of the donor and the ammonium groups of the acceptor. ^1^H NMR and X-ray diffraction data confirm the structure of D**·**A complexes. Complexes (*E*)-**1·**(*Z*)-**2** and (*E*)-**1·**(*E*)-**2** undergo the intrasupramolecular electron transfer upon excitation, which occurs efficiently even in a low-temperature glassy matrix of *n*-PrCN at 77 K. The absorption and luminescence parameters of these isomeric complexes show a significant variation in efficiency of the back electron transfer depending on the particular isomer, which is confirmed by transient absorption spectroscopy. The positions of the nitrogen atoms in the acceptor component of the complex has almost two orders of magnitude greater effect on the rate of this process than the involvement of either *Z-* or *E-*isomer of this component. In all probability, the major role in the efficiency of the back electron transfer belongs to the proximity of positions and the degree of overlap of HOMO and LUMO of the relaxed S_1r_(ET) and non-relaxed S_0_ states of the complex.

## Figures and Tables

**Table 1:** Stability constants and spectral characteristics of CT bands of (*E*)-**1** complexes with (*Z*)-**2**, (*E*)-**2**, and (*E*)-**3** ^a^.

Complex	log*K*_1_ (log*K*_2_)		*λ*_a_^max^/nm	*ε*_a_^max^/ M^−1^ cm^−1^
	Spectrophoto-Metric Titration	^1^H NMR Titration		
(*E*)-**1·**(*Z*)-**2**	8.48 ^b^	8.2	<400	–
(*E*)-**1·**(*E*)-**2**	8.67 ^c^	8.5	405 ^c,d^	430 ^c^
(*E*)-**1·**(*E*)-**3**	9.08 ^c^	8.9	502 ^c^	390 ^c^
(*E*)-**1·**(*Z*)-**2·**(*E*)-**1**	–	(0.6)	<400	–
(*E*)-**1·**(*E*)-**2·**(*E*)-**1**	(2.18) ^c^	(2.02) ^c^	416 ^c,d^	790 ^c^
(*E*)-**1·**(*E*)-**3·**(*E*)-**1**	(3.20) ^c^	(3.27) ^c^	519 ^c^	1020 ^c^

^a^ MeCN, 22 °C (for spectrophotometric data); MeCN-*d*_3_, 25 °C (for ^1^H NMR data). Stability constants, *K*_1_ and *K*_2_ (M^−1^), are measured to within ±30%. ^b^ Competitive spectrofluorimetric titration. ^c^ From Ref. [18]. ^d^ CT band as a shoulder.

**Table 2:** Spectral characteristics of compounds **1–3** and their complexes (without CT bands) ^a^.

Compound	*λ*_a_^max^/nm	*ε*_a_^max^/M^−1^cm^−1^	*λ*_f_^max^/nm	Stokes Shift/nm	Δ*λ*_f_^max^/nm 293–77 K (in *n*-PrCN)	*ϕ*_f_
(*E*)-**1** ^b^	336	37,500	386	50	4	0.3
(*E*)-**1·C10** ^c^	334	36,900	383	49	4	0.27
(*Z*)-**2**	278	16,080	400	112	–	0.067
(*E*)-**2**	284	27,350	393	109	–	0.218
(*E*)-**3** ^d^	321	44,000	369	48	–	0.02
(*E*)-**1·**(*Z*)-**2**	332	32,180	407	75	1.5	0.006
(*E*)-**1·**(*E*)-**2**	318	44,160	388	70	2	0.011
(*E*)-**1·**(*E*)-**3** ^d^	321	65,500	–	–	–	<10^−4^

^a^ MeCN, 293 K. ^b^ From Ref. [18] except for Δ*λ*_f_^max^ (293–77 K). ^c^ From Ref. [21] except for Δ*λ*_f_^max^ (293–77 K). ^d^ From Ref. [16].

**Table 3:** Spectral kinetic parameters of the TA spectra of complexes and their components in MeCN at 25° C.

Compound	*λ*_TA_^max^/nm	*τ*_1_/fs ^b^	*τ*_2_/ps ^b^	*τ*_3_/ns ^b^
(*E*)-**1** ^a^	561	69	–	~1.1
(*Z*)-**2**	–	250 ± 70	–	–
(*E*)-**2**	483 → 466	130 ± 20	–	2.9 ± 0.1
(*E*)-**1·C10**	563 → 573	45 ± 1	–	0.69 ± 0.04
(*E*)-**1·**(*Z*)-**2**	504	95 ± 10	16 ± 0.1	–
(*E*)-**1·**(*E*)-**2**	477	71 ± 3	36 ± 0.4	–
(*E*)-**1·**(*E*)-**3** ^a^	500	150	0.295	5.36 × 10^−4^

^a^ From Ref. [16], *λ*_TA_^max^ is in the range of 0.4–1.7 ps, *λ*_exc_ = 308 nm, 70 fs laser pulse. ^b^ *τ*_1_, *τ*_2_ are characteristic times of TA band buildup and decay for the compound. For (*E*)-**1·**(*E*)-**3**: *τ*_1_, *τ*_2_, *τ*_3_ are characteristic times related to the population of the LE state of the complex, internal conversion from high-lying excited states to lower CT state, and deactivation of the lower CT state via the back ET reaction, respectively.

**Table 4:** Geometrical characteristics of complexes: distance between the centers of masses of the donor and the acceptor (*l*_C_), range of distances from atoms of the donor to the acceptor atoms located most closely (*l*_min(D–A)_), dihedral angles between the planes of two pyridine residues of the acceptor (*θ*_AA_) and two benzene rings of the donor (*θ*_DD_), dihedral angles between the planes of the pyridine residue of the acceptor and the most closely located benzene ring of the donor (*θ*_AD_, *θ*_AD2_), and energy gaps between the S_0_–S_1_(ET) and S_1r_(ET)–S_0_ states ^a^.

Complex		*l*_C_/Å	*l*_min(D–A)_/Å	*θ*_AA_/°	*θ*_DD_/°	*θ*_AD_/°	*θ*_AD2_/°	∆*E*_S0/S1(ET)_ ∆*E*_S1r(ET)/S0_ /eV
(*E*)-**1·**(*Z*)-**2**	S_0_	4.1	3.4–4.3	54	3	65	22	3.2
	S_1_	3.9	3.2–4.1	41	10	52	21	2.1
(*E*)-**1·**(*E*)-**2**	S_0_	3.6	3.5–4.0	2	14	7	7	2.7
	S_1_	3.3	3.3–3.8	10	5	7	7	1.9
(*E*)-**1·**(*Z*)-**3**	S_0_	4.6	3.4–4.7	52	11	25	38	2.6
	S_1_	4.2	3.4–4.4	38	14	24	28	1.4
(*E*)-**1·**(*E*)-**3**	S_0_	3.8	3.4–4.2	7	8	1	8	2.3
	S_1_	3.9	3.2–4.0	7	7	4	7	1.3

^a^ **∆***E* values were calculated for the corresponding model complexes (see, for example, Figure 8): ∆*E*_S0/S1(ET)_ was found for the conformation of the molecule in the S_0_ state and corresponds to the excitation energy; ∆*E*_S1r(ET)/S0_ refers to the conformation optimal in the S_1_ state and corresponds to the emission energy.

**Table 5:** Crystal data and structure refinement details for (*E*)-**1·**8C_2_H_7_Br_2_N and [(*E*)-**1·**(*Z*)-**2**]**·**0.15 MeCN**·**1.275 H_2_O.

Structure	(*E*)-1·8C_2_H_7_Br_2_N	[(*E*)-1·(*Z*)-2] 0.15 MeCN·1.275 H_2_O
Formula	C_50_H_104_Br_16_N_8_O_12_	C_52_._3_H_79_Cl_4_N_4_._15_O_29_._275_
Formula weight, g·mol^−1^	2287.97	1376.09
Crystal system	monoclinic	triclinic
Space group	*P*2_1_/*n*	$P\;\bar{1}$
*a*, Å	16.7010 (3)	12.8857 (6)
*b*, Å	8.1317 (2)	15.8062 (7)
*c*, Å	28.8097 (6)	34.0819 (15)
*α*, °	90	78.271 (1)
*β*, °	91.738 (1)	79.560 (2)
*γ*, °	90	68.616 (1)
Volume, Å^3^	3910.77 (14)	6272.6 (5)
*Z*	2	4
*ρ*_calc_, g cm^−3^	1.943	1.457
*F* (000)	2232	2896
*μ* (Mo-K_α_), mm^−1^	8.243	0.280
Crystal size, mm	0.22 × 0.12 × 0.04	0.12 × 0.02 × 0.02
Temperature, K	120 (2)	100 (2)
2*θ* range for data collection, °	2.83 to 58.00	4.53 to 52.87
Index ranges	−22 ≤ *h* ≤ 19, −10 ≤ *k* ≤ 11, −39 ≤ *l* ≤ 39	−16 ≤ *h* ≤ 16, −19 ≤ *k* ≤ 19, −42 ≤ *l* ≤ 42
Collected reflections	32037	79617
Independent reflections	10,358 [*R*_int_ = 0.0674]	25,715 [*R*_int_ = 0.0903]
Reflections with *I* > 2*σ* (*I*)	6766	14,088
Data/restraints/parameters	10,358/7/402	25,715/1552/1904
Final *R* indices [*I* > 2*σ* (*I*)]	*R*_1_ = 0.0400, *wR*_2_ = 0.0693	*R*_1_ = 0.0736, *wR*_2_ = 0.1502
Final *R* indices [all data]	*R*_1_ = 0.0833, *wR*_2_ = 0.0769	*R*_1_ = 0.1507, *wR*_2_ = 0.1812
Goodness-of-fit on *F*^2^	0.942	1.025
Largest diff. peak/hole (max/min), ē Å^−3^	1.463/−1.060	0.763/−0.478

## Data Availability

The time-dependent absorption spectroscopy files are available from the respective author upon reasonable request.

## Supplementary Materials

The following supporting information can be downloaded at: https://www.mdpi.com/article/10.3390/molecules30193920/s1, Figures S1–S5. ^1^H NMR and ^13^C NMR spectra of *E*-2, (*E*)-**1·**(*Z*)-**2**, and *Z*-2; Figure S6. Stack packing in structure [(*E*)-**1·**(*Z*)-**2**]**·**0.15MeCN**·**1.275H_2_O; Figures S7 and S8. Structures of D·A complexes in the S_0_ and the S_1_ states; Table S1. Calculated energies (*E*) and oscillator strengths (*f*) of S_0_ → S*_n_* transitions of D·A complexes.
