# Supplementary material for: Highly Stable Supramolecular Donor–Acceptor Complexes Involving (Z)-, (E)-di(3-pyridyl)ethylene Derivatives as Weak Acceptors: Structure—Property Relationships
Source: Molecules. 2025 Sep 29;30(19):3920. doi: 10.3390/molecules30193920 (PMC12525814; doi:10.3390/molecules30193920)
Supplement: Supplementary file 1 [file molecules-30-03920-s001.zip › molecules-3878863-supplementary.pdf]

Supplementary Information

Highly Stable Supramolecular Donor–Acceptor Complexes Involving (*Z*)-, (*E*)-di(3-pyridyl)ethylene Derivatives as Weak Acceptors: Structure—Property Relationships

Artem I. Vedernikov [1], Valeriy V. Volchkov [2,*], Mikhail N. Khimich [2], Mikhail Y. Mel'nikov [2], Fedor E. Gostev [3], Ivan V. Shelaev [3], Victor A. Nadtochenko [3], Lyudmila G. Kuz'mina [4], Judith A. K. Howard [5], Asya A. Efremova [1], Mikhail V. Rusalov [1] and Sergey P. Gromov [1,2,*]

[1] NRC "Kurchatov Institute", Kurchatov Complex of Crystallography and Photonics, Photochemistry Center, 119421 Moscow, Russia
[2] Department of Chemistry, M. V. Lomonosov Moscow State University, 119991 Moscow, Russia
[3] N. N. Semenov Federal Research Center for Chemical Physics, Russian Academy of Sciences, 119991 Moscow, Russia
[4] N. S. Kurnakov Institute of General and Inorganic Chemistry, Russian Academy of Sciences, 119991 Moscow, Russia
[5] Department of Chemistry, Durham University, Durham DH1 3LE, UK; j.a.k.howard@durham.ac.uk
* Correspondence: volchkov_vv@mail.ru (V.V.V.); spgromov@mail.ru (S.P.G.)

CONTENT

Figures S1–S5. ^{1}H and ^{13}C NMR spectra of (*E*)-**2**, (*E*)-**1**·(*Z*)-**2**, and (*Z*)-**2** 2
Figure S6. Stack packing in structure [(*E*)-**1**·(*Z*)-**2**]·0.15MeCN·1.275H_2O 4
Figures S7, S8. Structures of D·A complexes in the S_0 and the S_1 states 5,6
Table S1. Calculated energies (*E*) and oscillator strengths (*f*) of $S_0 \rightarrow S_n$ transitions of D·A complexes. 6

^{1}H and ^{13}C NMR Spectra

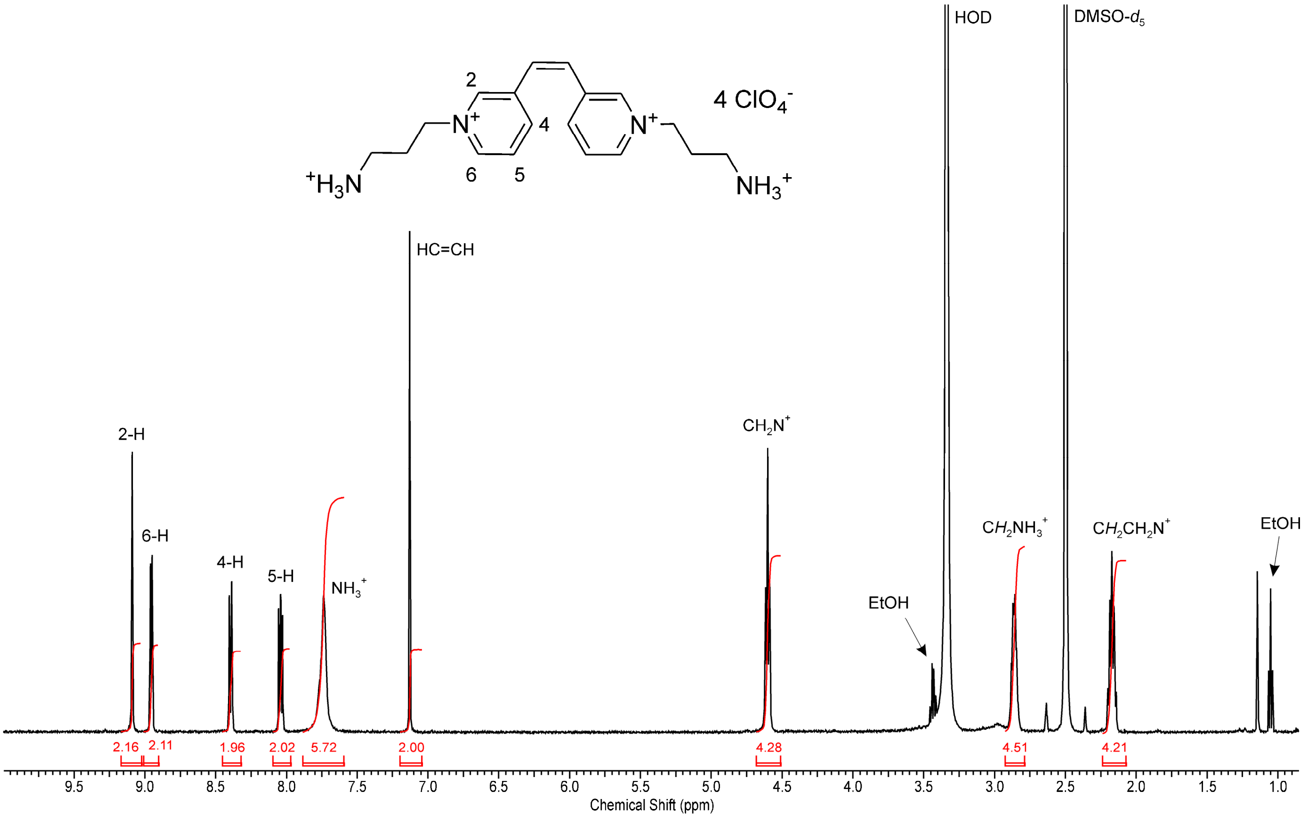

Figure S1. ^{1}H NMR spectrum of compound (Z)-**2** (500.13 MHz, DMSO-d_6, 30 °C).

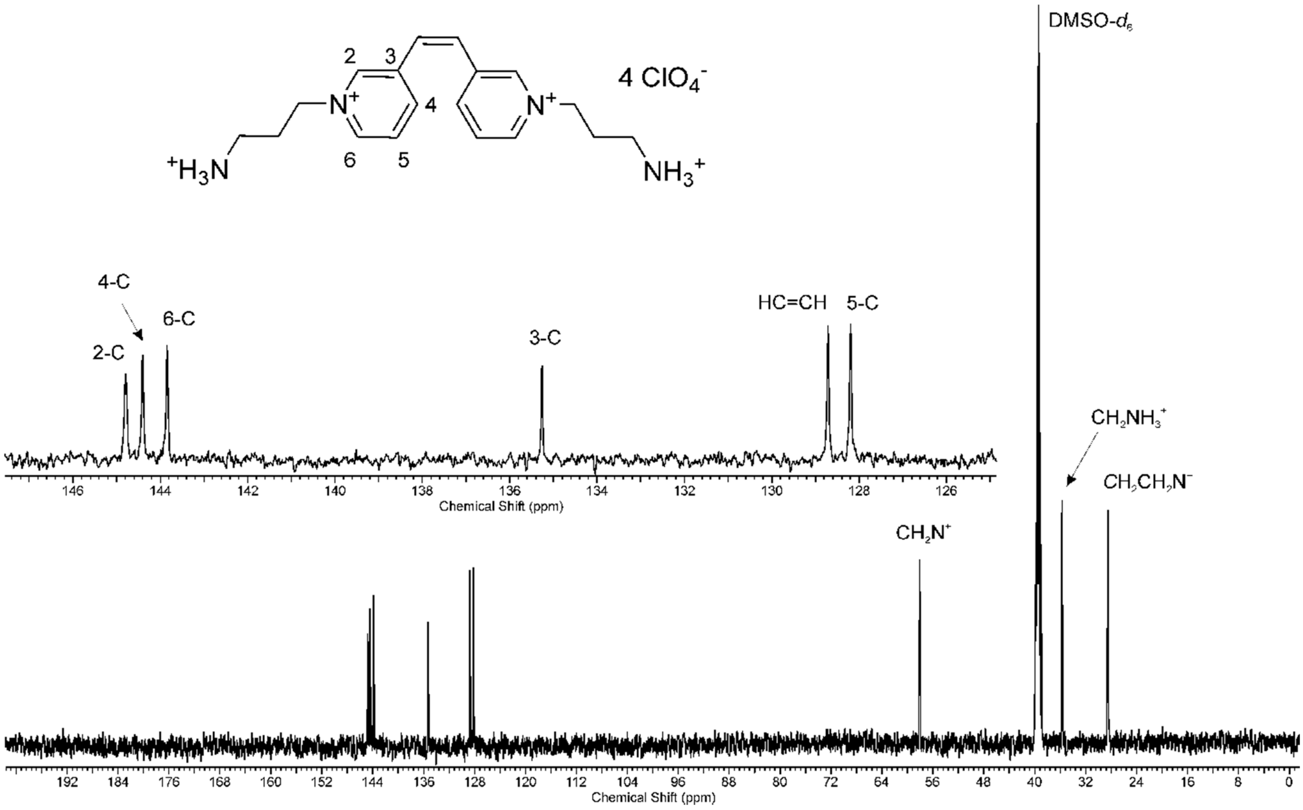

Figure S2. ^{13}C NMR spectrum of compound (Z)-**2** (125.76 MHz, DMSO-d_6, 26 °C).

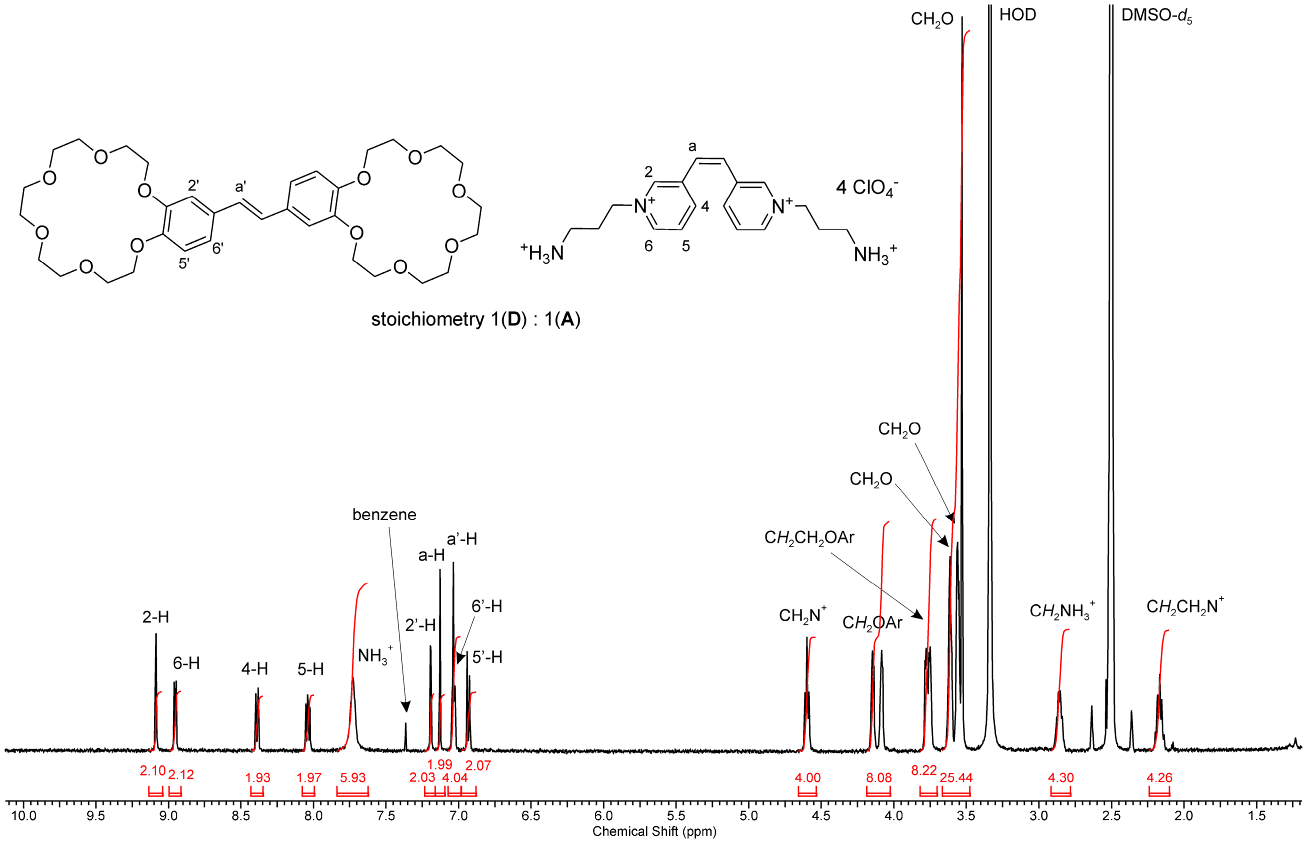

Figure S3. ¹H NMR spectrum of complex (*E*)-**1**·(*Z*)-**2**, which was obtained by crystallization (500.13 MHz, DMSO-*d*6, 25 °C). In DMSO-*d*6, the complex is destroyed to form a mixture of free compounds (*E*)-**1** and (*Z*)-**2**.

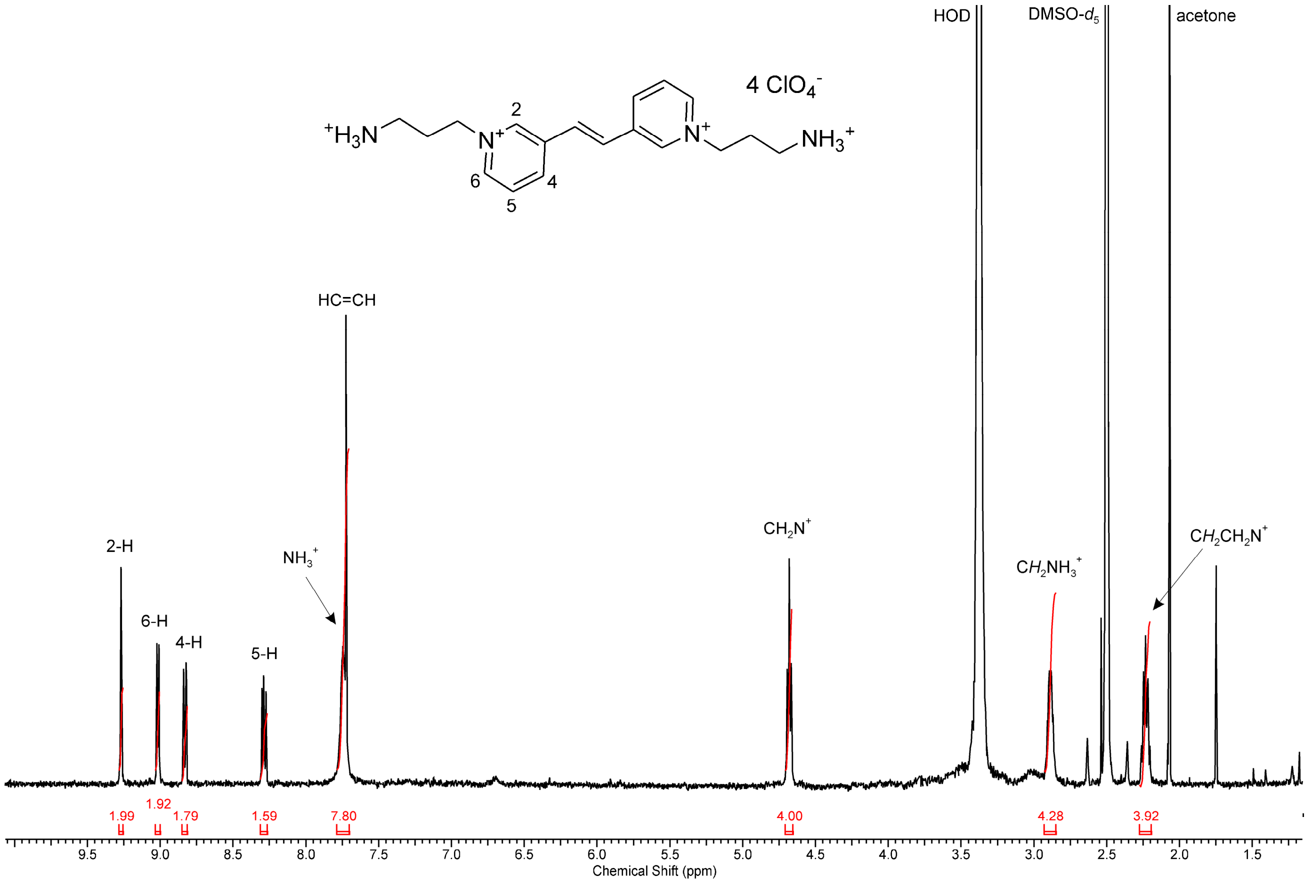

Figure S4. ¹H NMR spectrum of compound (*E*)-**2** (500.13 MHz, DMSO-*d*6, 25 °C).

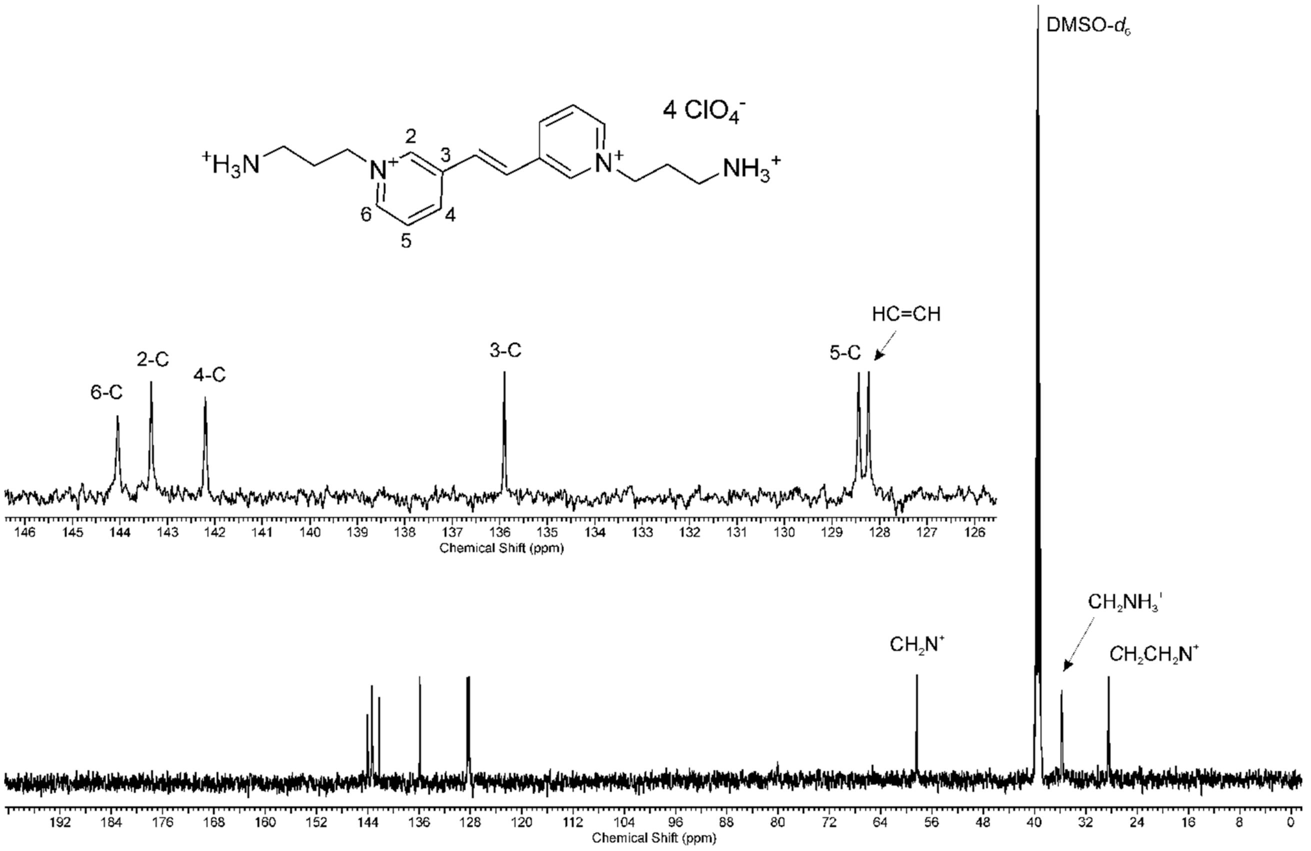

Figure S5. ^{13}C NMR spectrum of compound (*E*)-**2** (125.76 MHz, DMSO-*d*6, 25 °C).

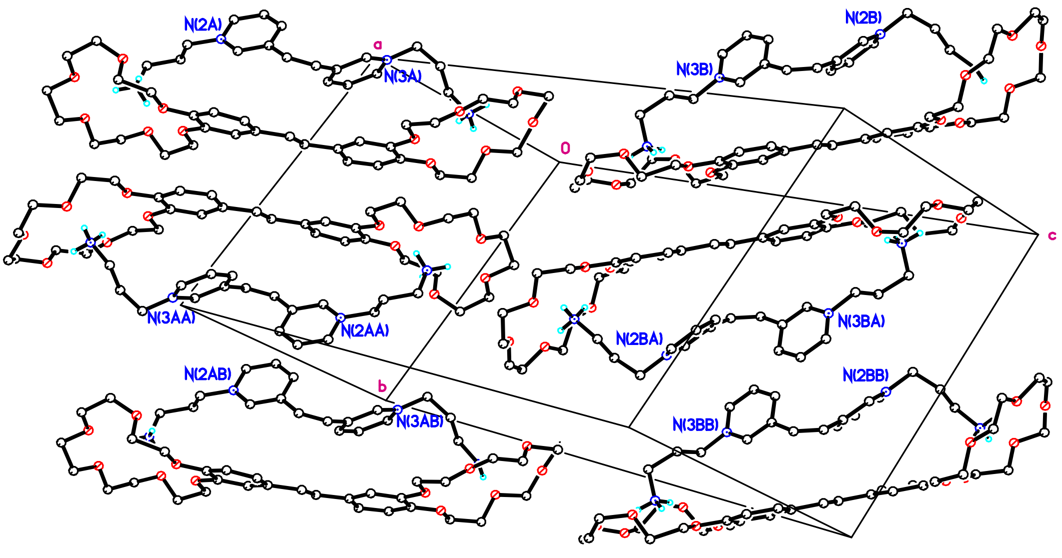

Figure S6. Stack packing in structure [(*E*)-**1**·(*Z*)-**2**]·0.15MeCN·1.275H_2O. The disorders of the crown ether moieties, most of the hydrogen atoms, perchlorate anions, and molecules of solvation are not shown for clarity.

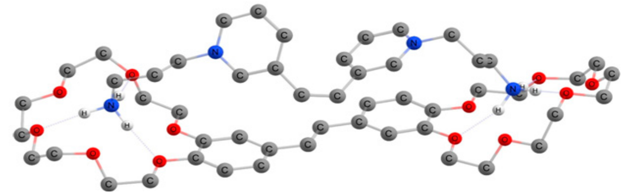

(*E*)-**1**·(*Z*)-**2**

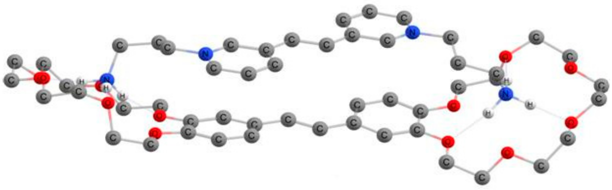

(*E*)-**1**·(*E*)-**2**

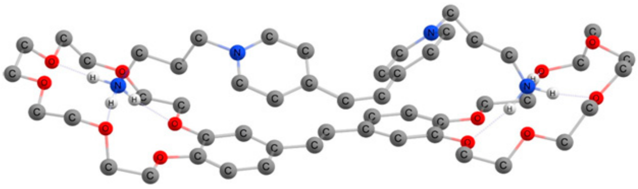

(*E*)-**1**·(*Z*)-**3**

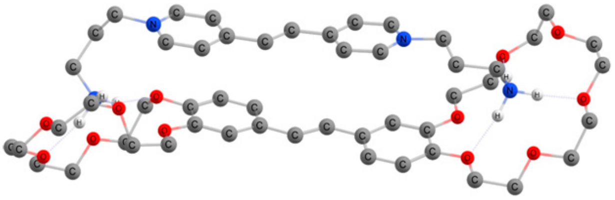

(*E*)-**1**·(*E*)-**3**

Figure S7. Calculated structures of D·A complexes in the S_0 state.

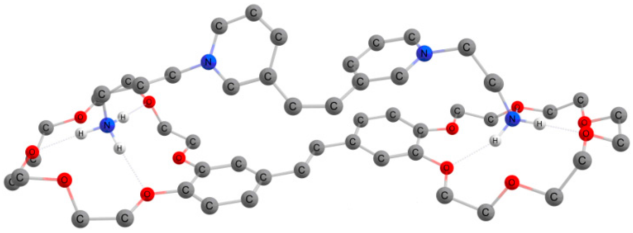

(*E*)-**1**·(*Z*)-**2**

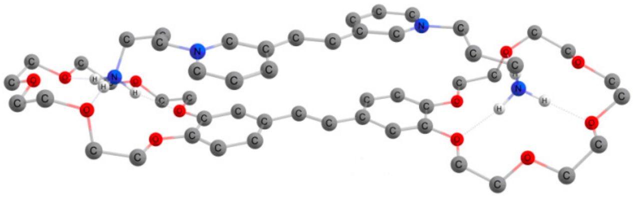

(*E*)-**1**·(*E*)-**2**

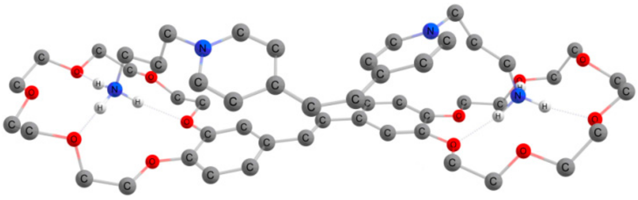

(*E*)-**1**·(*Z*)-**3**

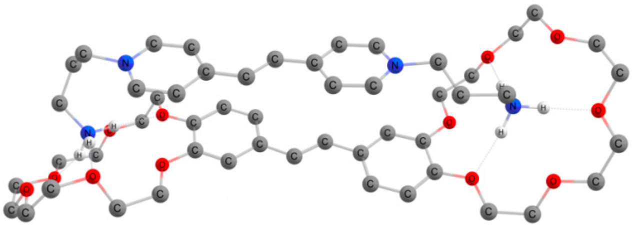

(*E*)-**1**·(*E*)-**3**

Figure S8. Calculated structures of D·A complexes in the S_1 state.

Table S1. Calculated energies (*E*) and oscillator strengths (*f*) of $S_0 \rightarrow S_n$ transitions of D·A complexes.

Complex		$S_0 \rightarrow S_1$	$S_0 \rightarrow S_2$	$S_0 \rightarrow S_3$	$S_0 \rightarrow S_4$	$S_0 \rightarrow S_5$	$S_0 \rightarrow S_6$	$S_0 \rightarrow S_7$	$S_0 \rightarrow S_8$
(*E*)-**1**·(*Z*)-**2**	*E*, eV	3.14	3.59	3.71	3.80	4.24	4.40	4.48	4.70
	f	0.08	0.05	0.04	1.27	0.03	0.16	0.02	0.11
(*E*)-**1**·(*E*)-**2**	*E*, eV	2.72	3.31	3.61	3.89	3.90	4.13	4.33	4.48
	f	0.001	0	0.05	0.36	0.02	1.21	0.32	0.001
(*E*)-**1**·(*Z*)-**3**	*E*, eV	2.35	3.20	3.77	3.81	4.04	4.05	4.37	4.41
	f	0.026	0.07	0.03	0.53	0.22	1.08	0.008	0.25
(*E*)-**1**·(*E*)-**3**	*E*, eV	2.65	3.54	3.85	3.96	4.09	4.25	4.26	4.50
	f	0.003	0.02	0.63	0.29	0.07	0.64	0.16	0.02
